# The differential production cross section of the $\phi$(1020) meson in $\sqrt{s}$ = 7 TeV $pp$ collisions measured with the ATLAS detector

**DOI:** 10.1140/epjc/s10052-014-2895-2

**Published:** 2014-07-01

**Authors:** The ATLAS Collaboration, G. Aad, T. Abajyan, B. Abbott, J. Abdallah, S. Abdel Khalek, A. A. Abdelalim, O. Abdinov, R. Aben, B. Abi, M. Abolins, O. S. AbouZeid, H. Abramowicz, H. Abreu, B. S. Acharya, L. Adamczyk, D. L. Adams, T. N. Addy, J. Adelman, S. Adomeit, P. Adragna, T. Adye, S. Aefsky, J. A. Aguilar-Saavedra, M. Agustoni, M. Aharrouche, S. P. Ahlen, F. Ahles, A. Ahmad, M. Ahsan, G. Aielli, T. P. A. Åkesson, G. Akimoto, A. V. Akimov, M. S. Alam, M. A. Alam, J. Albert, S. Albrand, M. Aleksa, I. N. Aleksandrov, F. Alessandria, C. Alexa, G. Alexander, G. Alexandre, T. Alexopoulos, M. Alhroob, M. Aliev, G. Alimonti, J. Alison, B. M. M. Allbrooke, P. P. Allport, S. E. Allwood-Spiers, J. Almond, A. Aloisio, R. Alon, A. Alonso, F. Alonso, A. Altheimer, B. Alvarez Gonzalez, M. G. Alviggi, K. Amako, C. Amelung, V. V. Ammosov, S. P. Amor Dos Santos, A. Amorim, N. Amram, C. Anastopoulos, L. S. Ancu, N. Andari, T. Andeen, C. F. Anders, G. Anders, K. J. Anderson, A. Andreazza, V. Andrei, M. -L. Andrieux, X. S. Anduaga, S. Angelidakis, P. Anger, A. Angerami, F. Anghinolfi, A. Anisenkov, N. Anjos, A. Annovi, A. Antonaki, M. Antonelli, A. Antonov, J. Antos, F. Anulli, M. Aoki, S. Aoun, L. Aperio Bella, R. Apolle, G. Arabidze, I. Aracena, Y. Arai, A. T. H. Arce, S. Arfaoui, J. -F. Arguin, S. Argyropoulos, E. Arik, M. Arik, A. J. Armbruster, O. Arnaez, V. Arnal, C. Arnault, A. Artamonov, G. Artoni, D. Arutinov, S. Asai, S. Ask, B. Åsman, L. Asquith, K. Assamagan, A. Astbury, M. Atkinson, B. Aubert, E. Auge, K. Augsten, M. Aurousseau, G. Avolio, R. Avramidou, D. Axen, G. Azuelos, Y. Azuma, M. A. Baak, G. Baccaglioni, C. Bacci, A. M. Bach, H. Bachacou, K. Bachas, M. Backes, M. Backhaus, J. Backus Mayes, E. Badescu, P. Bagnaia, S. Bahinipati, Y. Bai, D. C. Bailey, T. Bain, J. T. Baines, O. K. Baker, M. D. Baker, S. Baker, P. Balek, E. Banas, P. Banerjee, Sw. Banerjee, D. Banfi, A. Bangert, V. Bansal, H. S. Bansil, L. Barak, S. P. Baranov, A. Barbaro Galtieri, T. Barber, E. L. Barberio, D. Barberis, M. Barbero, D. Y. Bardin, T. Barillari, M. Barisonzi, T. Barklow, N. Barlow, B. M. Barnett, R. M. Barnett, A. Baroncelli, G. Barone, A. J. Barr, F. Barreiro, J. Barreiro Guimarães da Costa, P. Barrillon, R. Bartoldus, A. E. Barton, V. Bartsch, A. Basye, R. L. Bates, L. Batkova, J. R. Batley, A. Battaglia, M. Battistin, F. Bauer, H. S. Bawa, S. Beale, T. Beau, P. H. Beauchemin, R. Beccherle, P. Bechtle, H. P. Beck, A. K. Becker, S. Becker, M. Beckingham, K. H. Becks, A. J. Beddall, A. Beddall, S. Bedikian, V. A. Bednyakov, C. P. Bee, L. J. Beemster, M. Begel, S. Behar Harpaz, P. K. Behera, M. Beimforde, C. Belanger-Champagne, P. J. Bell, W. H. Bell, G. Bella, L. Bellagamba, M. Bellomo, A. Belloni, O. Beloborodova, K. Belotskiy, O. Beltramello, O. Benary, D. Benchekroun, K. Bendtz, N. Benekos, Y. Benhammou, E. Benhar Noccioli, J. A. Benitez Garcia, D. P. Benjamin, M. Benoit, J. R. Bensinger, K. Benslama, S. Bentvelsen, D. Berge, E. Bergeaas Kuutmann, N. Berger, F. Berghaus, E. Berglund, J. Beringer, P. Bernat, R. Bernhard, C. Bernius, T. Berry, C. Bertella, A. Bertin, F. Bertolucci, M. I. Besana, G. J. Besjes, N. Besson, S. Bethke, W. Bhimji, R. M. Bianchi, L. Bianchini, M. Bianco, O. Biebel, S. P. Bieniek, K. Bierwagen, J. Biesiada, M. Biglietti, H. Bilokon, M. Bindi, S. Binet, A. Bingul, C. Bini, C. Biscarat, B. Bittner, C. W. Black, K. M. Black, R. E. Blair, J. -B. Blanchard, G. Blanchot, T. Blazek, I. Bloch, C. Blocker, J. Blocki, A. Blondel, W. Blum, U. Blumenschein, G. J. Bobbink, V. B. Bobrovnikov, S. S. Bocchetta, A. Bocci, C. R. Boddy, M. Boehler, J. Boek, N. Boelaert, J. A. Bogaerts, A. Bogdanchikov, A. Bogouch, C. Bohm, J. Bohm, V. Boisvert, T. Bold, V. Boldea, N. M. Bolnet, M. Bomben, M. Bona, M. Boonekamp, S. Bordoni, C. Borer, A. Borisov, G. Borissov, I. Borjanovic, M. Borri, S. Borroni, J. Bortfeldt, V. Bortolotto, K. Bos, D. Boscherini, M. Bosman, H. Boterenbrood, J. Bouchami, J. Boudreau, E. V. Bouhova-Thacker, D. Boumediene, C. Bourdarios, N. Bousson, A. Boveia, J. Boyd, I. R. Boyko, I. Bozovic-Jelisavcic, J. Bracinik, P. Branchini, A. Brandt, G. Brandt, O. Brandt, U. Bratzler, B. Brau, J. E. Brau, H. M. Braun, S. F. Brazzale, B. Brelier, J. Bremer, K. Brendlinger, R. Brenner, S. Bressler, D. Britton, F. M. Brochu, I. Brock, R. Brock, F. Broggi, C. Bromberg, J. Bronner, G. Brooijmans, T. Brooks, W. K. Brooks, G. Brown, H. Brown, P. A. Bruckman de Renstrom, D. Bruncko, R. Bruneliere, S. Brunet, A. Bruni, G. Bruni, M. Bruschi, T. Buanes, Q. Buat, F. Bucci, J. Buchanan, P. Buchholz, R. M. Buckingham, A. G. Buckley, S. I. Buda, I. A. Budagov, B. Budick, V. Büscher, L. Bugge, O. Bulekov, A. C. Bundock, M. Bunse, T. Buran, H. Burckhart, S. Burdin, T. Burgess, S. Burke, E. Busato, P. Bussey, C. P. Buszello, B. Butler, J. M. Butler, C. M. Buttar, J. M. Butterworth, W. Buttinger, M. Byszewski, S. Cabrera Urbán, D. Caforio, O. Cakir, P. Calafiura, G. Calderini, P. Calfayan, R. Calkins, L. P. Caloba, R. Caloi, D. Calvet, S. Calvet, R. Camacho Toro, P. Camarri, D. Cameron, L. M. Caminada, R. Caminal Armadans, S. Campana, M. Campanelli, V. Canale, F. Canelli, A. Canepa, J. Cantero, R. Cantrill, L. Capasso, M. D. M. Capeans Garrido, I. Caprini, M. Caprini, D. Capriotti, M. Capua, R. Caputo, R. Cardarelli, T. Carli, G. Carlino, L. Carminati, B. Caron, S. Caron, E. Carquin, G. D. Carrillo-Montoya, A. A. Carter, J. R. Carter, J. Carvalho, D. Casadei, M. P. Casado, M. Cascella, C. Caso, A. M. Castaneda Hernandez, E. Castaneda-Miranda, V. Castillo Gimenez, N. F. Castro, G. Cataldi, P. Catastini, A. Catinaccio, J. R. Catmore, A. Cattai, G. Cattani, S. Caughron, V. Cavaliere, P. Cavalleri, D. Cavalli, M. Cavalli-Sforza, V. Cavasinni, F. Ceradini, A. S. Cerqueira, A. Cerri, L. Cerrito, F. Cerutti, S. A. Cetin, A. Chafaq, D. Chakraborty, I. Chalupkova, K. Chan, P. Chang, B. Chapleau, J. D. Chapman, J. W. Chapman, E. Chareyre, D. G. Charlton, V. Chavda, C. A. Chavez Barajas, S. Cheatham, S. Chekanov, S. V. Chekulaev, G. A. Chelkov, M. A. Chelstowska, C. Chen, H. Chen, S. Chen, X. Chen, Y. Chen, Y. Cheng, A. Cheplakov, R. Cherkaoui El Moursli, V. Chernyatin, E. Cheu, S. L. Cheung, L. Chevalier, G. Chiefari, L. Chikovani, J. T. Childers, A. Chilingarov, G. Chiodini, A. S. Chisholm, R. T. Chislett, A. Chitan, M. V. Chizhov, G. Choudalakis, S. Chouridou, I. A. Christidi, A. Christov, D. Chromek-Burckhart, M. L. Chu, J. Chudoba, G. Ciapetti, A. K. Ciftci, R. Ciftci, D. Cinca, V. Cindro, C. Ciocca, A. Ciocio, M. Cirilli, P. Cirkovic, Z. H. Citron, M. Citterio, M. Ciubancan, A. Clark, P. J. Clark, R. N. Clarke, W. Cleland, J. C. Clemens, B. Clement, C. Clement, Y. Coadou, M. Cobal, A. Coccaro, J. Cochran, L. Coffey, J. G. Cogan, J. Coggeshall, E. Cogneras, J. Colas, S. Cole, A. P. Colijn, N. J. Collins, C. Collins-Tooth, J. Collot, T. Colombo, G. Colon, G. Compostella, P. Conde Muiño, E. Coniavitis, M. C. Conidi, S. M. Consonni, V. Consorti, S. Constantinescu, C. Conta, G. Conti, F. Conventi, M. Cooke, B. D. Cooper, A. M. Cooper-Sarkar, K. Copic, T. Cornelissen, M. Corradi, F. Corriveau, A. Cortes-Gonzalez, G. Cortiana, G. Costa, M. J. Costa, D. Costanzo, D. Côté, L. Courneyea, G. Cowan, C. Cowden, B. E. Cox, K. Cranmer, F. Crescioli, M. Cristinziani, G. Crosetti, S. Crépé-Renaudin, C.-M. Cuciuc, C. Cuenca Almenar, T. Cuhadar Donszelmann, J. Cummings, M. Curatolo, C. J. Curtis, C. Cuthbert, P. Cwetanski, H. Czirr, P. Czodrowski, Z. Czyczula, S. D’Auria, M. D’Onofrio, A. D’Orazio, M. J. Da Cunha Sargedas De Sousa, C. Da Via, W. Dabrowski, A. Dafinca, T. Dai, C. Dallapiccola, M. Dam, M. Dameri, D. S. Damiani, H. O. Danielsson, V. Dao, G. Darbo, G. L. Darlea, J. A. Dassoulas, W. Davey, T. Davidek, N. Davidson, R. Davidson, E. Davies, M. Davies, O. Davignon, A. R. Davison, Y. Davygora, E. Dawe, I. Dawson, R. K. Daya-Ishmukhametova, K. De, R. de Asmundis, S. De Castro, S. De Cecco, J. de Graat, N. De Groot, P. de Jong, C. De La Taille, H. De la Torre, F. De Lorenzi, L. de Mora, L. De Nooij, D. De Pedis, A. De Salvo, U. De Sanctis, A. De Santo, J. B. De Vivie De Regie, G. De Zorzi, W. J. Dearnaley, R. Debbe, C. Debenedetti, B. Dechenaux, D. V. Dedovich, J. Degenhardt, J. Del Peso, T. Del Prete, T. Delemontex, M. Deliyergiyev, A. Dell’Acqua, L. Dell’Asta, M. Della Pietra, D. della Volpe, M. Delmastro, P. A. Delsart, C. Deluca, S. Demers, M. Demichev, B. Demirkoz, S. P. Denisov, D. Derendarz, J. E. Derkaoui, F. Derue, P. Dervan, K. Desch, E. Devetak, P. O. Deviveiros, A. Dewhurst, B. DeWilde, S. Dhaliwal, R. Dhullipudi, A. Di Ciaccio, L. Di Ciaccio, C. Di Donato, A. Di Girolamo, B. Di Girolamo, S. Di Luise, A. Di Mattia, B. Di Micco, R. Di Nardo, A. Di Simone, R. Di Sipio, M. A. Diaz, E. B. Diehl, J. Dietrich, T. A. Dietzsch, S. Diglio, K. Dindar Yagci, J. Dingfelder, F. Dinut, C. Dionisi, P. Dita, S. Dita, F. Dittus, F. Djama, T. Djobava, M. A. B. do Vale, A. Do Valle Wemans, T. K. O. Doan, M. Dobbs, D. Dobos, E. Dobson, J. Dodd, C. Doglioni, T. Doherty, Y. Doi, J. Dolejsi, I. Dolenc, Z. Dolezal, B. A. Dolgoshein, T. Dohmae, M. Donadelli, J. Donini, J. Dopke, A. Doria, A. Dos Anjos, A. Dotti, M. T. Dova, A. D. Doxiadis, A. T. Doyle, N. Dressnandt, M. Dris, J. Dubbert, S. Dube, E. Duchovni, G. Duckeck, D. Duda, A. Dudarev, F. Dudziak, M. Dührssen, I. P. Duerdoth, L. Duflot, M. -A. Dufour, L. Duguid, M. Dunford, H. Duran Yildiz, R. Duxfield, M. Dwuznik, M. Düren, W. L. Ebenstein, J. Ebke, S. Eckweiler, K. Edmonds, W. Edson, C. A. Edwards, N. C. Edwards, W. Ehrenfeld, T. Eifert, G. Eigen, K. Einsweiler, E. Eisenhandler, T. Ekelof, M. El Kacimi, M. Ellert, S. Elles, F. Ellinghaus, K. Ellis, N. Ellis, J. Elmsheuser, M. Elsing, D. Emeliyanov, R. Engelmann, A. Engl, B. Epp, J. Erdmann, A. Ereditato, D. Eriksson, J. Ernst, M. Ernst, J. Ernwein, D. Errede, S. Errede, E. Ertel, M. Escalier, H. Esch, C. Escobar, X. Espinal Curull, B. Esposito, F. Etienne, A. I. Etienvre, E. Etzion, D. Evangelakou, H. Evans, L. Fabbri, C. Fabre, R. M. Fakhrutdinov, S. Falciano, Y. Fang, M. Fanti, A. Farbin, A. Farilla, J. Farley, T. Farooque, S. Farrell, S. M. Farrington, P. Farthouat, F. Fassi, P. Fassnacht, D. Fassouliotis, B. Fatholahzadeh, A. Favareto, L. Fayard, S. Fazio, R. Febbraro, P. Federic, O. L. Fedin, W. Fedorko, M. Fehling-Kaschek, L. Feligioni, C. Feng, E. J. Feng, A. B. Fenyuk, J. Ferencei, W. Fernando, S. Ferrag, J. Ferrando, V. Ferrara, A. Ferrari, P. Ferrari, R. Ferrari, D. E. Ferreira de Lima, A. Ferrer, D. Ferrere, C. Ferretti, A. Ferretto Parodi, M. Fiascaris, F. Fiedler, A. Filipčič, F. Filthaut, M. Fincke-Keeler, M. C. N. Fiolhais, L. Fiorini, A. Firan, G. Fischer, M. J. Fisher, M. Flechl, I. Fleck, J. Fleckner, P. Fleischmann, S. Fleischmann, T. Flick, A. Floderus, L. R. Flores Castillo, M. J. Flowerdew, T. Fonseca Martin, A. Formica, A. Forti, D. Fortin, D. Fournier, A. J. Fowler, H. Fox, P. Francavilla, M. Franchini, S. Franchino, D. Francis, T. Frank, M. Franklin, S. Franz, M. Fraternali, S. Fratina, S. T. French, C. Friedrich, F. Friedrich, R. Froeschl, D. Froidevaux, J. A. Frost, C. Fukunaga, E. Fullana Torregrosa, B. G. Fulsom, J. Fuster, C. Gabaldon, O. Gabizon, T. Gadfort, S. Gadomski, G. Gagliardi, P. Gagnon, C. Galea, B. Galhardo, E. J. Gallas, V. Gallo, B. J. Gallop, P. Gallus, K. K. Gan, Y. S. Gao, A. Gaponenko, F. Garberson, M. Garcia-Sciveres, C. García, J. E. García Navarro, R. W. Gardner, N. Garelli, H. Garitaonandia, V. Garonne, C. Gatti, G. Gaudio, B. Gaur, L. Gauthier, P. Gauzzi, I. L. Gavrilenko, C. Gay, G. Gaycken, E. N. Gazis, P. Ge, Z. Gecse, C. N. P. Gee, D. A. A. Geerts, Ch. Geich-Gimbel, K. Gellerstedt, C. Gemme, A. Gemmell, M. H. Genest, S. Gentile, M. George, S. George, P. Gerlach, A. Gershon, C. Geweniger, H. Ghazlane, N. Ghodbane, B. Giacobbe, S. Giagu, V. Giakoumopoulou, V. Giangiobbe, F. Gianotti, B. Gibbard, A. Gibson, S. M. Gibson, M. Gilchriese, D. Gillberg, A. R. Gillman, D. M. Gingrich, J. Ginzburg, N. Giokaris, M. P. Giordani, R. Giordano, F. M. Giorgi, P. Giovannini, P. F. Giraud, D. Giugni, M. Giunta, B. K. Gjelsten, L. K. Gladilin, C. Glasman, J. Glatzer, A. Glazov, K. W. Glitza, G. L. Glonti, J. R. Goddard, J. Godfrey, J. Godlewski, M. Goebel, T. Göpfert, C. Goeringer, C. Gössling, S. Goldfarb, T. Golling, A. Gomes, L. S. Gomez Fajardo, R. Gonçalo, J. Goncalves Pinto Firmino Da Costa, L. Gonella, S. González de la Hoz, G. Gonzalez Parra, M. L. Gonzalez Silva, S. Gonzalez-Sevilla, J. J. Goodson, L. Goossens, P. A. Gorbounov, H. A. Gordon, I. Gorelov, G. Gorfine, B. Gorini, E. Gorini, A. Gorišek, E. Gornicki, A. T. Goshaw, M. Gosselink, M. I. Gostkin, I. Gough Eschrich, M. Gouighri, D. Goujdami, M. P. Goulette, A. G. Goussiou, C. Goy, S. Gozpinar, I. Grabowska-Bold, P. Grafström, K. -J. Grahn, E. Gramstad, F. Grancagnolo, S. Grancagnolo, V. Grassi, V. Gratchev, N. Grau, H. M. Gray, J. A. Gray, E. Graziani, O. G. Grebenyuk, T. Greenshaw, Z. D. Greenwood, K. Gregersen, I. M. Gregor, P. Grenier, J. Griffiths, N. Grigalashvili, A. A. Grillo, S. Grinstein, Ph. Gris, Y. V. Grishkevich, J. -F. Grivaz, E. Gross, J. Grosse-Knetter, J. Groth-Jensen, K. Grybel, D. Guest, C. Guicheney, E. Guido, S. Guindon, U. Gul, J. Gunther, B. Guo, J. Guo, P. Gutierrez, N. Guttman, O. Gutzwiller, C. Guyot, C. Gwenlan, C. B. Gwilliam, A. Haas, S. Haas, C. Haber, H. K. Hadavand, D. R. Hadley, P. Haefner, F. Hahn, Z. Hajduk, H. Hakobyan, D. Hall, K. Hamacher, P. Hamal, K. Hamano, M. Hamer, A. Hamilton, S. Hamilton, L. Han, K. Hanagaki, K. Hanawa, M. Hance, C. Handel, P. Hanke, J. R. Hansen, J. B. Hansen, J. D. Hansen, P. H. Hansen, P. Hansson, K. Hara, T. Harenberg, S. Harkusha, D. Harper, R. D. Harrington, O. M. Harris, J. Hartert, F. Hartjes, T. Haruyama, A. Harvey, S. Hasegawa, Y. Hasegawa, S. Hassani, S. Haug, M. Hauschild, R. Hauser, M. Havranek, C. M. Hawkes, R. J. Hawkings, A. D. Hawkins, T. Hayakawa, T. Hayashi, D. Hayden, C. P. Hays, H. S. Hayward, S. J. Haywood, S. J. Head, V. Hedberg, L. Heelan, S. Heim, B. Heinemann, S. Heisterkamp, L. Helary, C. Heller, M. Heller, S. Hellman, D. Hellmich, C. Helsens, R. C. W. Henderson, M. Henke, A. Henrichs, A. M. Henriques Correia, S. Henrot-Versille, C. Hensel, T. Henß, C. M. Hernandez, Y. Hernández Jiménez, R. Herrberg, G. Herten, R. Hertenberger, L. Hervas, G. G. Hesketh, N. P. Hessey, E. Higón-Rodriguez, J. C. Hill, K. H. Hiller, S. Hillert, S. J. Hillier, I. Hinchliffe, E. Hines, M. Hirose, F. Hirsch, D. Hirschbuehl, J. Hobbs, N. Hod, M. C. Hodgkinson, P. Hodgson, A. Hoecker, M. R. Hoeferkamp, J. Hoffman, D. Hoffmann, M. Hohlfeld, M. Holder, S. O. Holmgren, T. Holy, J. L. Holzbauer, T. M. Hong, L. Hooft van Huysduynen, S. Horner, J-Y. Hostachy, S. Hou, A. Hoummada, J. Howard, J. Howarth, I. Hristova, J. Hrivnac, T. Hryn’ova, P. J. Hsu, S.-C. Hsu, D. Hu, Z. Hubacek, F. Hubaut, F. Huegging, A. Huettmann, T. B. Huffman, E. W. Hughes, G. Hughes, M. Huhtinen, M. Hurwitz, N. Huseynov, J. Huston, J. Huth, G. Iacobucci, G. Iakovidis, M. Ibbotson, I. Ibragimov, L. Iconomidou-Fayard, J. Idarraga, P. Iengo, O. Igonkina, Y. Ikegami, M. Ikeno, D. Iliadis, N. Ilic, T. Ince, P. Ioannou, M. Iodice, K. Iordanidou, V. Ippolito, A. Irles Quiles, C. Isaksson, M. Ishino, M. Ishitsuka, R. Ishmukhametov, C. Issever, S. Istin, A. V. Ivashin, W. Iwanski, H. Iwasaki, J. M. Izen, V. Izzo, B. Jackson, J. N. Jackson, P. Jackson, M. R. Jaekel, V. Jain, K. Jakobs, S. Jakobsen, T. Jakoubek, J. Jakubek, D. O. Jamin, D. K. Jana, E. Jansen, H. Jansen, J. Janssen, A. Jantsch, M. Janus, R. C. Jared, G. Jarlskog, L. Jeanty, I. Jen-La Plante, D. Jennens, P. Jenni, A. E. Loevschall-Jensen, P. Jež, S. Jézéquel, M. K. Jha, H. Ji, W. Ji, J. Jia, Y. Jiang, M. Jimenez Belenguer, S. Jin, O. Jinnouchi, M. D. Joergensen, D. Joffe, M. Johansen, K. E. Johansson, P. Johansson, S. Johnert, K. A. Johns, K. Jon-And, G. Jones, R. W. L. Jones, T. J. Jones, C. Joram, P. M. Jorge, K. D. Joshi, J. Jovicevic, T. Jovin, X. Ju, C. A. Jung, R. M. Jungst, V. Juranek, P. Jussel, A. Juste Rozas, S. Kabana, M. Kaci, A. Kaczmarska, P. Kadlecik, M. Kado, H. Kagan, M. Kagan, E. Kajomovitz, S. Kalinin, L. V. Kalinovskaya, S. Kama, N. Kanaya, M. Kaneda, S. Kaneti, T. Kanno, V. A. Kantserov, J. Kanzaki, B. Kaplan, A. Kapliy, J. Kaplon, D. Kar, M. Karagounis, K. Karakostas, M. Karnevskiy, V. Kartvelishvili, A. N. Karyukhin, L. Kashif, G. Kasieczka, R. D. Kass, A. Kastanas, M. Kataoka, Y. Kataoka, E. Katsoufis, J. Katzy, V. Kaushik, K. Kawagoe, T. Kawamoto, G. Kawamura, M. S. Kayl, S. Kazama, V. A. Kazanin, M. Y. Kazarinov, R. Keeler, P. T. Keener, R. Kehoe, M. Keil, G. D. Kekelidze, J. S. Keller, M. Kenyon, O. Kepka, N. Kerschen, B. P. Kerševan, S. Kersten, K. Kessoku, J. Keung, F. Khalil-zada, H. Khandanyan, A. Khanov, D. Kharchenko, A. Khodinov, A. Khomich, T. J. Khoo, G. Khoriauli, A. Khoroshilov, V. Khovanskiy, E. Khramov, J. Khubua, H. Kim, S. H. Kim, N. Kimura, O. Kind, B. T. King, M. King, R. S. B. King, J. Kirk, A. E. Kiryunin, T. Kishimoto, D. Kisielewska, T. Kitamura, T. Kittelmann, K. Kiuchi, E. Kladiva, M. Klein, U. Klein, K. Kleinknecht, M. Klemetti, A. Klier, P. Klimek, A. Klimentov, R. Klingenberg, J. A. Klinger, E. B. Klinkby, T. Klioutchnikova, P. F. Klok, S. Klous, E.-E. Kluge, T. Kluge, P. Kluit, S. Kluth, E. Kneringer, E. B. F. G. Knoops, A. Knue, B. R. Ko, T. Kobayashi, M. Kobel, M. Kocian, P. Kodys, K. Köneke, A. C. König, S. Koenig, L. Köpke, F. Koetsveld, P. Koevesarki, T. Koffas, E. Koffeman, L. A. Kogan, S. Kohlmann, F. Kohn, Z. Kohout, T. Kohriki, T. Koi, G. M. Kolachev, H. Kolanoski, V. Kolesnikov, I. Koletsou, J. Koll, A. A. Komar, Y. Komori, T. Kondo, T. Kono, A. I. Kononov, R. Konoplich, N. Konstantinidis, R. Kopeliansky, S. Koperny, K. Korcyl, K. Kordas, A. Korn, A. Korol, I. Korolkov, E. V. Korolkova, V. A. Korotkov, O. Kortner, S. Kortner, V. V. Kostyukhin, S. Kotov, V. M. Kotov, A. Kotwal, C. Kourkoumelis, V. Kouskoura, A. Koutsman, R. Kowalewski, T. Z. Kowalski, W. Kozanecki, A. S. Kozhin, V. Kral, V. A. Kramarenko, G. Kramberger, M. W. Krasny, A. Krasznahorkay, J. K. Kraus, S. Kreiss, F. Krejci, J. Kretzschmar, N. Krieger, P. Krieger, K. Kroeninger, H. Kroha, J. Kroll, J. Kroseberg, J. Krstic, U. Kruchonak, H. Krüger, T. Kruker, N. Krumnack, Z. V. Krumshteyn, M. K. Kruse, T. Kubota, S. Kuday, S. Kuehn, A. Kugel, T. Kuhl, D. Kuhn, V. Kukhtin, Y. Kulchitsky, S. Kuleshov, C. Kummer, M. Kuna, J. Kunkle, A. Kupco, H. Kurashige, M. Kurata, Y. A. Kurochkin, V. Kus, E. S. Kuwertz, M. Kuze, J. Kvita, R. Kwee, A. La Rosa, L. La Rotonda, L. Labarga, J. Labbe, S. Lablak, C. Lacasta, F. Lacava, J. Lacey, H. Lacker, D. Lacour, V. R. Lacuesta, E. Ladygin, R. Lafaye, B. Laforge, T. Lagouri, S. Lai, E. Laisne, L. Lambourne, C. L. Lampen, W. Lampl, E. Lancon, U. Landgraf, M. P. J. Landon, V. S. Lang, C. Lange, A. J. Lankford, F. Lanni, K. Lantzsch, S. Laplace, C. Lapoire, J. F. Laporte, T. Lari, A. Larner, M. Lassnig, P. Laurelli, V. Lavorini, W. Lavrijsen, P. Laycock, O. Le Dortz, E. Le Guirriec, E. Le Menedeu, T. LeCompte, F. Ledroit-Guillon, H. Lee, J. S. H. Lee, S. C. Lee, L. Lee, M. Lefebvre, M. Legendre, F. Legger, C. Leggett, M. Lehmacher, G. Lehmann Miotto, A. G. Leister, M. A. L. Leite, R. Leitner, D. Lellouch, B. Lemmer, V. Lendermann, K. J. C. Leney, T. Lenz, G. Lenzen, B. Lenzi, K. Leonhardt, S. Leontsinis, F. Lepold, C. Leroy, J-R. Lessard, C. G. Lester, C. M. Lester, J. Levêque, D. Levin, L. J. Levinson, A. Lewis, G. H. Lewis, A. M. Leyko, M. Leyton, B. Li, B. Li, H. Li, H. L. Li, S. Li, X. Li, Z. Liang, H. Liao, B. Liberti, P. Lichard, M. Lichtnecker, K. Lie, W. Liebig, C. Limbach, A. Limosani, M. Limper, S. C. Lin, F. Linde, J. T. Linnemann, E. Lipeles, A. Lipniacka, T. M. Liss, D. Lissauer, A. Lister, A. M. Litke, C. Liu, D. Liu, H. Liu, J. B. Liu, L. Liu, M. Liu, Y. Liu, M. Livan, S. S. A. Livermore, A. Lleres, J. Llorente Merino, S. L. Lloyd, E. Lobodzinska, P. Loch, W. S. Lockman, T. Loddenkoetter, F. K. Loebinger, A. Loginov, C. W. Loh, T. Lohse, K. Lohwasser, M. Lokajicek, V. P. Lombardo, R. E. Long, L. Lopes, D. Lopez Mateos, J. Lorenz, N. Lorenzo Martinez, M. Losada, P. Loscutoff, F. Lo Sterzo, M. J. Losty, X. Lou, A. Lounis, K. F. Loureiro, J. Love, P. A. Love, A. J. Lowe, F. Lu, H. J. Lubatti, C. Luci, A. Lucotte, A. Ludwig, D. Ludwig, I. Ludwig, J. Ludwig, F. Luehring, G. Luijckx, W. Lukas, L. Luminari, E. Lund, B. Lund-Jensen, B. Lundberg, J. Lundberg, O. Lundberg, J. Lundquist, M. Lungwitz, D. Lynn, E. Lytken, H. Ma, L. L. Ma, G. Maccarrone, A. Macchiolo, B. Maček, J. Machado Miguens, D. Macina, R. Mackeprang, R. J. Madaras, H. J. Maddocks, W. F. Mader, R. Maenner, T. Maeno, P. Mättig, S. Mättig, L. Magnoni, E. Magradze, K. Mahboubi, J. Mahlstedt, S. Mahmoud, G. Mahout, C. Maiani, C. Maidantchik, A. Maio, S. Majewski, Y. Makida, N. Makovec, P. Mal, B. Malaescu, Pa. Malecki, P. Malecki, V. P. Maleev, F. Malek, U. Mallik, D. Malon, C. Malone, S. Maltezos, V. Malyshev, S. Malyukov, R. Mameghani, J. Mamuzic, A. Manabe, L. Mandelli, I. Mandić, R. Mandrysch, J. Maneira, A. Manfredini, L. Manhaes de Andrade Filho, J. A. Manjarres Ramos, A. Mann, P. M. Manning, A. Manousakis-Katsikakis, B. Mansoulie, A. Mapelli, L. Mapelli, L. March, J. F. Marchand, F. Marchese, G. Marchiori, M. Marcisovsky, C. P. Marino, F. Marroquim, Z. Marshall, L. F. Marti, S. Marti-Garcia, B. Martin, B. Martin, J. P. Martin, T. A. Martin, V. J. Martin, B. Martin dit Latour, S. Martin-Haugh, M. Martinez, V. Martinez Outschoorn, A. C. Martyniuk, M. Marx, F. Marzano, A. Marzin, L. Masetti, T. Mashimo, R. Mashinistov, J. Masik, A. L. Maslennikov, I. Massa, G. Massaro, N. Massol, P. Mastrandrea, A. Mastroberardino, T. Masubuchi, P. Matricon, H. Matsunaga, T. Matsushita, C. Mattravers, J. Maurer, S. J. Maxfield, D. A. Maximov, A. Mayne, R. Mazini, M. Mazur, L. Mazzaferro, M. Mazzanti, J. Mc Donald, S. P. Mc Kee, A. McCarn, R. L. McCarthy, T. G. McCarthy, N. A. McCubbin, K. W. McFarlane, J. A. Mcfayden, G. Mchedlidze, T. Mclaughlan, S. J. McMahon, R. A. McPherson, A. Meade, J. Mechnich, M. Mechtel, M. Medinnis, S. Meehan, R. Meera-Lebbai, T. Meguro, S. Mehlhase, A. Mehta, K. Meier, B. Meirose, C. Melachrinos, B. R. Mellado Garcia, F. Meloni, L. Mendoza Navas, Z. Meng, A. Mengarelli, S. Menke, E. Meoni, K. M. Mercurio, P. Mermod, L. Merola, C. Meroni, F. S. Merritt, H. Merritt, A. Messina, J. Metcalfe, A. S. Mete, C. Meyer, C. Meyer, J-P. Meyer, J. Meyer, J. Meyer, S. Michal, L. Micu, R. P. Middleton, S. Migas, L. Mijović, G. Mikenberg, M. Mikestikova, M. Mikuž, D. W. Miller, R. J. Miller, W. J. Mills, C. Mills, A. Milov, D. A. Milstead, D. Milstein, A. A. Minaenko, M. Miñano Moya, I. A. Minashvili, A. I. Mincer, B. Mindur, M. Mineev, Y. Ming, L. M. Mir, G. Mirabelli, J. Mitrevski, V. A. Mitsou, S. Mitsui, P. S. Miyagawa, J. U. Mjörnmark, T. Moa, V. Moeller, K. Mönig, N. Möser, S. Mohapatra, W. Mohr, R. Moles-Valls, A. Molfetas, J. Monk, E. Monnier, J. Montejo Berlingen, F. Monticelli, S. Monzani, R. W. Moore, G. F. Moorhead, C. Mora Herrera, A. Moraes, N. Morange, J. Morel, G. Morello, D. Moreno, M. Moreno Llácer, P. Morettini, M. Morgenstern, M. Morii, A. K. Morley, G. Mornacchi, J. D. Morris, L. Morvaj, H. G. Moser, M. Mosidze, J. Moss, R. Mount, E. Mountricha, S. V. Mouraviev, E. J. W. Moyse, F. Mueller, J. Mueller, K. Mueller, T. A. Müller, T. Mueller, D. Muenstermann, Y. Munwes, W. J. Murray, I. Mussche, E. Musto, A. G. Myagkov, M. Myska, O. Nackenhorst, J. Nadal, K. Nagai, R. Nagai, K. Nagano, A. Nagarkar, Y. Nagasaka, M. Nagel, A. M. Nairz, Y. Nakahama, K. Nakamura, T. Nakamura, I. Nakano, G. Nanava, A. Napier, R. Narayan, M. Nash, T. Nattermann, T. Naumann, G. Navarro, H. A. Neal, P. Yu. Nechaeva, T. J. Neep, A. Negri, G. Negri, M. Negrini, S. Nektarijevic, A. Nelson, T. K. Nelson, S. Nemecek, P. Nemethy, A. A. Nepomuceno, M. Nessi, M. S. Neubauer, M. Neumann, A. Neusiedl, R. M. Neves, P. Nevski, F. M. Newcomer, P. R. Newman, V. Nguyen Thi Hong, R. B. Nickerson, R. Nicolaidou, B. Nicquevert, F. Niedercorn, J. Nielsen, N. Nikiforou, A. Nikiforov, V. Nikolaenko, I. Nikolic-Audit, K. Nikolics, K. Nikolopoulos, H. Nilsen, P. Nilsson, Y. Ninomiya, A. Nisati, R. Nisius, T. Nobe, L. Nodulman, M. Nomachi, I. Nomidis, S. Norberg, M. Nordberg, P. R. Norton, J. Novakova, M. Nozaki, L. Nozka, I. M. Nugent, A. -E. Nuncio-Quiroz, G. Nunes Hanninger, T. Nunnemann, E. Nurse, B. J. O’Brien, D. C. O’Neil, V. O’Shea, L. B. Oakes, F. G. Oakham, H. Oberlack, J. Ocariz, A. Ochi, S. Oda, S. Odaka, J. Odier, H. Ogren, A. Oh, S. H. Oh, C. C. Ohm, T. Ohshima, W. Okamura, H. Okawa, Y. Okumura, T. Okuyama, A. Olariu, A. G. Olchevski, S. A. Olivares Pino, M. Oliveira, D. Oliveira Damazio, E. Oliver Garcia, D. Olivito, A. Olszewski, J. Olszowska, A. Onofre, P. U. E. Onyisi, C. J. Oram, M. J. Oreglia, Y. Oren, D. Orestano, N. Orlando, I. Orlov, C. Oropeza Barrera, R. S. Orr, B. Osculati, R. Ospanov, C. Osuna, G. Otero y Garzon, J. P. Ottersbach, M. Ouchrif, E. A. Ouellette, F. Ould-Saada, A. Ouraou, Q. Ouyang, A. Ovcharova, M. Owen, S. Owen, V. E. Ozcan, N. Ozturk, A. Pacheco Pages, C. Padilla Aranda, S. Pagan Griso, E. Paganis, C. Pahl, F. Paige, P. Pais, K. Pajchel, G. Palacino, C. P. Paleari, S. Palestini, D. Pallin, A. Palma, J. D. Palmer, Y. B. Pan, E. Panagiotopoulou, J. G. Panduro Vazquez, P. Pani, N. Panikashvili, S. Panitkin, D. Pantea, A. Papadelis, Th. D. Papadopoulou, A. Paramonov, D. Paredes Hernandez, W. Park, M. A. Parker, F. Parodi, J. A. Parsons, U. Parzefall, S. Pashapour, E. Pasqualucci, S. Passaggio, A. Passeri, F. Pastore, Fr. Pastore, G. Pásztor, S. Pataraia, N. Patel, J. R. Pater, S. Patricelli, T. Pauly, M. Pecsy, S. Pedraza Lopez, M. I. Pedraza Morales, S. V. Peleganchuk, D. Pelikan, H. Peng, B. Penning, A. Penson, J. Penwell, M. Perantoni, K. Perez, T. Perez Cavalcanti, E. Perez Codina, M. T. Pérez García-Estañ, V. Perez Reale, L. Perini, H. Pernegger, R. Perrino, P. Perrodo, V. D. Peshekhonov, K. Peters, B. A. Petersen, J. Petersen, T. C. Petersen, E. Petit, A. Petridis, C. Petridou, E. Petrolo, F. Petrucci, D. Petschull, M. Petteni, R. Pezoa, A. Phan, P. W. Phillips, G. Piacquadio, A. Picazio, E. Piccaro, M. Piccinini, S. M. Piec, R. Piegaia, D. T. Pignotti, J. E. Pilcher, A. D. Pilkington, J. Pina, M. Pinamonti, A. Pinder, J. L. Pinfold, B. Pinto, C. Pizio, M. Plamondon, M.-A. Pleier, E. Plotnikova, A. Poblaguev, S. Poddar, F. Podlyski, L. Poggioli, D. Pohl, M. Pohl, G. Polesello, A. Policicchio, A. Polini, J. Poll, V. Polychronakos, D. Pomeroy, K. Pommès, L. Pontecorvo, B. G. Pope, G. A. Popeneciu, D. S. Popovic, A. Poppleton, X. Portell Bueso, G. E. Pospelov, S. Pospisil, I. N. Potrap, C. J. Potter, C. T. Potter, G. Poulard, J. Poveda, V. Pozdnyakov, R. Prabhu, P. Pralavorio, A. Pranko, S. Prasad, R. Pravahan, S. Prell, K. Pretzl, D. Price, J. Price, L. E. Price, D. Prieur, M. Primavera, K. Prokofiev, F. Prokoshin, S. Protopopescu, J. Proudfoot, X. Prudent, M. Przybycien, H. Przysiezniak, S. Psoroulas, E. Ptacek, E. Pueschel, J. Purdham, M. Purohit, P. Puzo, Y. Pylypchenko, J. Qian, A. Quadt, D. R. Quarrie, W. B. Quayle, F. Quinonez, M. Raas, V. Radeka, V. Radescu, P. Radloff, F. Ragusa, G. Rahal, A. M. Rahimi, D. Rahm, S. Rajagopalan, M. Rammensee, M. Rammes, A. S. Randle-Conde, K. Randrianarivony, F. Rauscher, T. C. Rave, M. Raymond, A. L. Read, D. M. Rebuzzi, A. Redelbach, G. Redlinger, R. Reece, K. Reeves, A. Reinsch, I. Reisinger, C. Rembser, Z. L. Ren, A. Renaud, M. Rescigno, S. Resconi, B. Resende, P. Reznicek, R. Rezvani, R. Richter, E. Richter-Was, M. Ridel, M. Rijpstra, M. Rijssenbeek, A. Rimoldi, L. Rinaldi, R. R. Rios, I. Riu, G. Rivoltella, F. Rizatdinova, E. Rizvi, S. H. Robertson, A. Robichaud-Veronneau, D. Robinson, J. E. M. Robinson, A. Robson, J. G. Rocha de Lima, C. Roda, D. Roda Dos Santos, A. Roe, S. Roe, O. Røhne, S. Rolli, A. Romaniouk, M. Romano, G. Romeo, E. Romero Adam, N. Rompotis, L. Roos, E. Ros, S. Rosati, K. Rosbach, A. Rose, M. Rose, G. A. Rosenbaum, E. I. Rosenberg, P. L. Rosendahl, O. Rosenthal, L. Rosselet, V. Rossetti, E. Rossi, L. P. Rossi, M. Rotaru, I. Roth, J. Rothberg, D. Rousseau, C. R. Royon, A. Rozanov, Y. Rozen, X. Ruan, F. Rubbo, I. Rubinskiy, N. Ruckstuhl, V. I. Rud, C. Rudolph, G. Rudolph, F. Rühr, A. Ruiz-Martinez, L. Rumyantsev, Z. Rurikova, N. A. Rusakovich, A. Ruschke, J. P. Rutherfoord, P. Ruzicka, Y. F. Ryabov, M. Rybar, G. Rybkin, N. C. Ryder, A. F. Saavedra, I. Sadeh, H. F -W. Sadrozinski, R. Sadykov, F. Safai Tehrani, H. Sakamoto, G. Salamanna, A. Salamon, M. Saleem, D. Salek, D. Salihagic, A. Salnikov, J. Salt, B. M. Salvachua Ferrando, D. Salvatore, F. Salvatore, A. Salvucci, A. Salzburger, D. Sampsonidis, B. H. Samset, A. Sanchez, V. Sanchez Martinez, H. Sandaker, H. G. Sander, M. P. Sanders, M. Sandhoff, T. Sandoval, C. Sandoval, R. Sandstroem, D. P. C. Sankey, A. Sansoni, C. Santamarina Rios, C. Santoni, R. Santonico, H. Santos, I. Santoyo Castillo, J. G. Saraiva, T. Sarangi, E. Sarkisyan-Grinbaum, F. Sarri, G. Sartisohn, O. Sasaki, Y. Sasaki, N. Sasao, I. Satsounkevitch, G. Sauvage, E. Sauvan, J. B. Sauvan, P. Savard, V. Savinov, D. O. Savu, L. Sawyer, D. H. Saxon, J. Saxon, C. Sbarra, A. Sbrizzi, D. A. Scannicchio, M. Scarcella, J. Schaarschmidt, P. Schacht, D. Schaefer, U. Schäfer, A. Schaelicke, S. Schaepe, S. Schaetzel, A. C. Schaffer, D. Schaile, R. D. Schamberger, A. G. Schamov, V. Scharf, V. A. Schegelsky, D. Scheirich, M. Schernau, M. I. Scherzer, C. Schiavi, J. Schieck, M. Schioppa, S. Schlenker, E. Schmidt, K. Schmieden, C. Schmitt, S. Schmitt, B. Schneider, U. Schnoor, L. Schoeffel, A. Schoening, A. L. S. Schorlemmer, M. Schott, D. Schouten, J. Schovancova, M. Schram, C. Schroeder, N. Schroer, M. J. Schultens, J. Schultes, H. -C. Schultz-Coulon, H. Schulz, M. Schumacher, B. A. Schumm, Ph. Schune, C. Schwanenberger, A. Schwartzman, Ph. Schwegler, Ph. Schwemling, R. Schwienhorst, R. Schwierz, J. Schwindling, T. Schwindt, M. Schwoerer, F. G. Sciacca, G. Sciolla, W. G. Scott, J. Searcy, G. Sedov, E. Sedykh, S. C. Seidel, A. Seiden, F. Seifert, J. M. Seixas, G. Sekhniaidze, S. J. Sekula, K. E. Selbach, D. M. Seliverstov, B. Sellden, G. Sellers, M. Seman, N. Semprini-Cesari, C. Serfon, L. Serin, L. Serkin, R. Seuster, H. Severini, A. Sfyrla, E. Shabalina, M. Shamim, L. Y. Shan, J. T. Shank, Q. T. Shao, M. Shapiro, P. B. Shatalov, K. Shaw, D. Sherman, P. Sherwood, S. Shimizu, M. Shimojima, T. Shin, M. Shiyakova, A. Shmeleva, M. J. Shochet, D. Short, S. Shrestha, E. Shulga, M. A. Shupe, P. Sicho, A. Sidoti, F. Siegert, DJ. Sijacki, O. Silbert, J. Silva, Y. Silver, D. Silverstein, S. B. Silverstein, V. Simak, O. Simard, Lj. Simic, S. Simion, E. Simioni, B. Simmons, R. Simoniello, M. Simonyan, P. Sinervo, N. B. Sinev, V. Sipica, G. Siragusa, A. Sircar, A. N. Sisakyan, S. Yu. Sivoklokov, J. Sjölin, T. B. Sjursen, L. A. Skinnari, H. P. Skottowe, K. Skovpen, P. Skubic, M. Slater, T. Slavicek, K. Sliwa, V. Smakhtin, B. H. Smart, L. Smestad, S. Yu. Smirnov, Y. Smirnov, L. N. Smirnova, O. Smirnova, B. C. Smith, D. Smith, K. M. Smith, M. Smizanska, K. Smolek, A. A. Snesarev, S. W. Snow, J. Snow, S. Snyder, R. Sobie, J. Sodomka, A. Soffer, C. A. Solans, M. Solar, J. Solc, E. Yu. Soldatov, U. Soldevila, E. Solfaroli Camillocci, A. A. Solodkov, O. V. Solovyanov, V. Solovyev, N. Soni, V. Sopko, B. Sopko, M. Sosebee, R. Soualah, A. Soukharev, S. Spagnolo, F. Spanò, R. Spighi, G. Spigo, R. Spiwoks, M. Spousta, T. Spreitzer, B. Spurlock, R. D. St. Denis, J. Stahlman, R. Stamen, E. Stanecka, R. W. Stanek, C. Stanescu, M. Stanescu-Bellu, M. M. Stanitzki, S. Stapnes, E. A. Starchenko, J. Stark, P. Staroba, P. Starovoitov, R. Staszewski, A. Staude, P. Stavina, G. Steele, P. Steinbach, P. Steinberg, I. Stekl, B. Stelzer, H. J. Stelzer, O. Stelzer-Chilton, H. Stenzel, S. Stern, G. A. Stewart, J. A. Stillings, M. C. Stockton, K. Stoerig, G. Stoicea, S. Stonjek, P. Strachota, A. R. Stradling, A. Straessner, J. Strandberg, S. Strandberg, A. Strandlie, M. Strang, E. Strauss, M. Strauss, P. Strizenec, R. Ströhmer, D. M. Strom, J. A. Strong, R. Stroynowski, B. Stugu, I. Stumer, J. Stupak, P. Sturm, N. A. Styles, D. A. Soh, D. Su, H S. Subramania, R. Subramaniam, A. Succurro, Y. Sugaya, C. Suhr, M. Suk, V. V. Sulin, S. Sultansoy, T. Sumida, X. Sun, J. E. Sundermann, K. Suruliz, G. Susinno, M. R. Sutton, Y. Suzuki, Y. Suzuki, M. Svatos, S. Swedish, I. Sykora, T. Sykora, J. Sánchez, D. Ta, K. Tackmann, A. Taffard, R. Tafirout, N. Taiblum, Y. Takahashi, H. Takai, R. Takashima, H. Takeda, T. Takeshita, Y. Takubo, M. Talby, A. Talyshev, M. C. Tamsett, K. G. Tan, J. Tanaka, R. Tanaka, S. Tanaka, S. Tanaka, A. J. Tanasijczuk, K. Tani, N. Tannoury, S. Tapprogge, D. Tardif, S. Tarem, F. Tarrade, G. F. Tartarelli, P. Tas, M. Tasevsky, E. Tassi, Y. Tayalati, C. Taylor, F. E. Taylor, G. N. Taylor, W. Taylor, M. Teinturier, F. A. Teischinger, M. Teixeira Dias Castanheira, P. Teixeira-Dias, K. K. Temming, H. Ten Kate, P. K. Teng, S. Terada, K. Terashi, J. Terron, M. Testa, R. J. Teuscher, J. Therhaag, T. Theveneaux-Pelzer, S. Thoma, J. P. Thomas, E. N. Thompson, P. D. Thompson, P. D. Thompson, A. S. Thompson, L. A. Thomsen, E. Thomson, M. Thomson, W. M. Thong, R. P. Thun, F. Tian, M. J. Tibbetts, T. Tic, V. O. Tikhomirov, Y. A. Tikhonov, S. Timoshenko, E. Tiouchichine, P. Tipton, S. Tisserant, T. Todorov, S. Todorova-Nova, B. Toggerson, J. Tojo, S. Tokár, K. Tokushuku, K. Tollefson, M. Tomoto, L. Tompkins, K. Toms, A. Tonoyan, C. Topfel, N. D. Topilin, E. Torrence, H. Torres, E. Torró Pastor, J. Toth, F. Touchard, D. R. Tovey, T. Trefzger, L. Tremblet, A. Tricoli, I. M. Trigger, S. Trincaz-Duvoid, M. F. Tripiana, N. Triplett, W. Trischuk, B. Trocmé, C. Troncon, M. Trottier-McDonald, P. True, M. Trzebinski, A. Trzupek, C. Tsarouchas, J. C-L. Tseng, M. Tsiakiris, P. V. Tsiareshka, D. Tsionou, G. Tsipolitis, S. Tsiskaridze, V. Tsiskaridze, E. G. Tskhadadze, I. I. Tsukerman, V. Tsulaia, J. -W. Tsung, S. Tsuno, D. Tsybychev, A. Tua, A. Tudorache, V. Tudorache, J. M. Tuggle, M. Turala, D. Turecek, I. Turk Cakir, E. Turlay, R. Turra, P. M. Tuts, A. Tykhonov, M. Tylmad, M. Tyndel, G. Tzanakos, K. Uchida, I. Ueda, R. Ueno, M. Ugland, M. Uhlenbrock, M. Uhrmacher, F. Ukegawa, G. Unal, A. Undrus, G. Unel, Y. Unno, D. Urbaniec, P. Urquijo, G. Usai, M. Uslenghi, L. Vacavant, V. Vacek, B. Vachon, S. Vahsen, J. Valenta, S. Valentinetti, A. Valero, S. Valkar, E. Valladolid Gallego, S. Vallecorsa, J. A. Valls Ferrer, R. Van Berg, P. C. Van Der Deijl, R. van der Geer, H. van der Graaf, R. Van Der Leeuw, E. van der Poel, D. van der Ster, N. van Eldik, P. van Gemmeren, I. van Vulpen, M. Vanadia, W. Vandelli, A. Vaniachine, P. Vankov, F. Vannucci, R. Vari, E. W. Varnes, T. Varol, D. Varouchas, A. Vartapetian, K. E. Varvell, V. I. Vassilakopoulos, F. Vazeille, T. Vazquez Schroeder, G. Vegni, J. J. Veillet, F. Veloso, R. Veness, S. Veneziano, A. Ventura, D. Ventura, M. Venturi, N. Venturi, V. Vercesi, M. Verducci, W. Verkerke, J. C. Vermeulen, A. Vest, M. C. Vetterli, I. Vichou, T. Vickey, O. E. Vickey Boeriu, G. H. A. Viehhauser, S. Viel, M. Villa, M. Villaplana Perez, E. Vilucchi, M. G. Vincter, E. Vinek, V. B. Vinogradov, M. Virchaux, J. Virzi, O. Vitells, M. Viti, I. Vivarelli, F. Vives Vaque, S. Vlachos, D. Vladoiu, M. Vlasak, A. Vogel, P. Vokac, G. Volpi, M. Volpi, G. Volpini, H. von der Schmitt, H. von Radziewski, E. von Toerne, V. Vorobel, V. Vorwerk, M. Vos, R. Voss, T. T. Voss, J. H. Vossebeld, N. Vranjes, M. Vranjes Milosavljevic, V. Vrba, M. Vreeswijk, T. Vu Anh, R. Vuillermet, I. Vukotic, W. Wagner, P. Wagner, H. Wahlen, S. Wahrmund, J. Wakabayashi, S. Walch, J. Walder, R. Walker, W. Walkowiak, R. Wall, P. Waller, B. Walsh, C. Wang, H. Wang, H. Wang, J. Wang, J. Wang, R. Wang, S. M. Wang, T. Wang, A. Warburton, C. P. Ward, D. R. Wardrope, M. Warsinsky, A. Washbrook, C. Wasicki, I. Watanabe, P. M. Watkins, A. T. Watson, I. J. Watson, M. F. Watson, G. Watts, S. Watts, A. T. Waugh, B. M. Waugh, M. S. Weber, J. S. Webster, A. R. Weidberg, P. Weigell, J. Weingarten, C. Weiser, P. S. Wells, T. Wenaus, D. Wendland, Z. Weng, T. Wengler, S. Wenig, N. Wermes, M. Werner, P. Werner, M. Werth, M. Wessels, J. Wetter, C. Weydert, K. Whalen, A. White, M. J. White, S. White, S. R. Whitehead, D. Whiteson, D. Whittington, F. Wicek, D. Wicke, F. J. Wickens, W. Wiedenmann, M. Wielers, P. Wienemann, C. Wiglesworth, L. A. M. Wiik-Fuchs, P. A. Wijeratne, A. Wildauer, M. A. Wildt, I. Wilhelm, H. G. Wilkens, J. Z. Will, E. Williams, H. H. Williams, W. Willis, S. Willocq, J. A. Wilson, M. G. Wilson, A. Wilson, I. Wingerter-Seez, S. Winkelmann, F. Winklmeier, M. Wittgen, S. J. Wollstadt, M. W. Wolter, H. Wolters, W. C. Wong, G. Wooden, B. K. Wosiek, J. Wotschack, M. J. Woudstra, K. W. Wozniak, K. Wraight, M. Wright, B. Wrona, S. L. Wu, X. Wu, Y. Wu, E. Wulf, B. M. Wynne, S. Xella, M. Xiao, S. Xie, C. Xu, D. Xu, L. Xu, B. Yabsley, S. Yacoob, M. Yamada, H. Yamaguchi, A. Yamamoto, K. Yamamoto, S. Yamamoto, T. Yamamura, T. Yamanaka, T. Yamazaki, Y. Yamazaki, Z. Yan, H. Yang, U. K. Yang, Y. Yang, Z. Yang, S. Yanush, L. Yao, Y. Yao, Y. Yasu, G. V. Ybeles Smit, J. Ye, S. Ye, M. Yilmaz, R. Yoosoofmiya, K. Yorita, R. Yoshida, K. Yoshihara, C. Young, C. J. Young, S. Youssef, D. Yu, J. Yu, J. Yu, L. Yuan, A. Yurkewicz, B. Zabinski, R. Zaidan, A. M. Zaitsev, Z. Zajacova, L. Zanello, D. Zanzi, A. Zaytsev, C. Zeitnitz, M. Zeman, A. Zemla, C. Zendler, O. Zenin, T. Ženiš, Z. Zinonos, D. Zerwas, G. Zevi della Porta, D. Zhang, H. Zhang, J. Zhang, X. Zhang, Z. Zhang, L. Zhao, Z. Zhao, A. Zhemchugov, J. Zhong, B. Zhou, N. Zhou, Y. Zhou, C. G. Zhu, H. Zhu, J. Zhu, Y. Zhu, X. Zhuang, V. Zhuravlov, A. Zibell, D. Zieminska, N. I. Zimin, R. Zimmermann, S. Zimmermann, S. Zimmermann, M. Ziolkowski, R. Zitoun, L. Živković, V. V. Zmouchko, G. Zobernig, A. Zoccoli, M. zur Nedden, V. Zutshi, L. Zwalinski

**Affiliations:** 1 School of Chemistry and Physics, University of Adelaide, Adelaide, Australia; 2 Physics Department, SUNY Albany, Albany, NY USA; 3 Department of Physics, University of Alberta, Edmonton, AB Canada; 4 Department of Physics, Ankara University, Ankara; Department of Physics, Dumlupinar University, Kutahya; Department of Physics, Gazi University, Ankara; Division of Physics, TOBB University of Economics and Technology, Ankara; Turkish Atomic Energy Authority, Ankara, Turkey; 5 LAPP, CNRS/IN2P3 and Université de Savoie, Annecy-le-Vieux, France; 6 High Energy Physics Division, Argonne National Laboratory, Argonne, IL USA; 7 Department of Physics, University of Arizona, Tucson, AZ USA; 8 Department of Physics, The University of Texas at Arlington, Arlington, TX USA; 9 Physics Department, University of Athens, Athens, Greece; 10 Physics Department, National Technical University of Athens, Zografou, Greece; 11 Institute of Physics, Azerbaijan Academy of Sciences, Baku, Azerbaijan; 12 Institut de Física d’Altes Energies and Departament de Física de la Universitat Autònoma de Barcelona and ICREA, Barcelona, Spain; 13 Institute of Physics, University of Belgrade, Belgrade; Vinca Institute of Nuclear Sciences, University of Belgrade, Belgrade, Serbia; 14 Department for Physics and Technology, University of Bergen, Bergen, Norway; 15 Physics Division, Lawrence Berkeley National Laboratory and University of California, Berkeley, CA USA; 16 Department of Physics, Humboldt University, Berlin, Germany; 17 Albert Einstein Center for Fundamental Physics and Laboratory for High Energy Physics, University of Bern, Bern, Switzerland; 18 School of Physics and Astronomy, University of Birmingham, Birmingham, UK; 19 Department of Physics, Bogazici University, Istanbul; Division of Physics, Dogus University, Istanbul; Department of Physics Engineering, Gaziantep University, Gaziantep; Department of Physics, Istanbul Technical University, Istanbul, Turkey; 20 INFN Sezione di Bologna; Dipartimento di Fisica, Università di Bologna, Bologna, Italy; 21 Physikalisches Institut, University of Bonn, Bonn, Germany; 22 Department of Physics, Boston University, Boston, MA USA; 23 Department of Physics, Brandeis University, Waltham, MA USA; 24 Universidade Federal do Rio De Janeiro COPPE/EE/IF, Rio de Janeiro; Federal University of Juiz de Fora (UFJF), Juiz de Fora; Federal University of Sao Joao del Rei (UFSJ), Sao Joao del Rei; Instituto de Fisica, Universidade de Sao Paulo, Sao Paulo, Brazil; 25 Physics Department, Brookhaven National Laboratory, Upton, NY USA; 26 National Institute of Physics and Nuclear Engineering, Bucharest; University Politehnica Bucharest, Bucharest; West University in Timisoara, Timisoara, Romania; 27 Departamento de Física, Universidad de Buenos Aires, Buenos Aires, Argentina; 28 Cavendish Laboratory, University of Cambridge, Cambridge, UK; 29 Department of Physics, Carleton University, Ottawa, ON Canada; 30 CERN, Geneva, Switzerland; 31 Enrico Fermi Institute, University of Chicago, Chicago, IL USA; 32 Departamento de Física, Pontificia Universidad Católica de Chile, Santiago; Departamento de Física, Universidad Técnica Federico Santa María, Valparaíso, Chile; 33 Institute of High Energy Physics, Chinese Academy of Sciences, Beijing; Department of Modern Physics, University of Science and Technology of China, Anhui; Department of Physics, Nanjing University, Jiangsu; School of Physics, Shandong University, Shandong, China; 34 Laboratoire de Physique Corpusculaire, Clermont Université and Université Blaise Pascal and CNRS/IN2P3, Clermont-Ferrand, France; 35 Nevis Laboratory, Columbia University, Irvington, NY USA; 36 Niels Bohr Institute, University of Copenhagen, Kobenhavn, Denmark; 37 INFN Gruppo Collegato di Cosenza; Dipartimento di Fisica, Università della Calabria, Arcavata di Rende, Italy; 38 AGH University of Science and Technology, Faculty of Physics and Applied Computer Science, Krakow, Poland; 39 The Henryk Niewodniczanski Institute of Nuclear Physics, Polish Academy of Sciences, Krakow, Poland; 40 Physics Department, Southern Methodist University, Dallas, TX USA; 41 Physics Department, University of Texas at Dallas, Richardson, TX USA; 42 DESY, Hamburg and Zeuthen, Germany; 43 Institut für Experimentelle Physik IV, Technische Universität Dortmund, Dortmund, Germany; 44 Institut für Kern- und Teilchenphysik, Technical University Dresden, Dresden, Germany; 45 Department of Physics, Duke University, Durham, NC USA; 46 SUPA, School of Physics and Astronomy, University of Edinburgh, Edinburgh, UK; 47 INFN Laboratori Nazionali di Frascati, Frascati, Italy; 48 Fakultät für Mathematik und Physik, Albert-Ludwigs-Universität, Freiburg, Germany; 49 Section de Physique, Université de Genève, Geneva, Switzerland; 50 INFN Sezione di Genova; Dipartimento di Fisica, Università di Genova, Genova, Italy; 51 E. Andronikashvili Institute of Physics, Iv. Javakhishvili Tbilisi State University, Tbilisi; High Energy Physics Institute, Tbilisi State University, Tbilisi, Georgia; 52 II Physikalisches Institut, Justus-Liebig-Universität Giessen, Giessen, Germany; 53 SUPA, School of Physics and Astronomy, University of Glasgow, Glasgow, UK; 54 II Physikalisches Institut, Georg-August-Universität, Göttingen, Germany; 55 Laboratoire de Physique Subatomique et de Cosmologie, Université Joseph Fourier and CNRS/IN2P3 and Institut National Polytechnique de Grenoble, Grenoble, France; 56 Department of Physics, Hampton University, Hampton, VA USA; 57 Laboratory for Particle Physics and Cosmology, Harvard University, Cambridge, MA USA; 58 Kirchhoff-Institut für Physik, Ruprecht-Karls-Universität Heidelberg, Heidelberg; Physikalisches Institut, Ruprecht-Karls-Universität Heidelberg, Heidelberg; ZITI Institut für technische Informatik, Ruprecht-Karls-Universität Heidelberg, Mannheim, Germany; 59 Faculty of Applied Information Science, Hiroshima Institute of Technology, Hiroshima, Japan; 60 Department of Physics, Indiana University, Bloomington, IN USA; 61 Institut für Astro- und Teilchenphysik, Leopold-Franzens-Universität, Innsbruck, Austria; 62 University of Iowa, Iowa City, IA USA; 63 Department of Physics and Astronomy, Iowa State University, Ames, IA USA; 64 Joint Institute for Nuclear Research, JINR Dubna, Dubna, Russia; 65 KEK, High Energy Accelerator Research Organization, Tsukuba, Japan; 66 Graduate School of Science, Kobe University, Kobe, Japan; 67 Faculty of Science, Kyoto University, Kyoto, Japan; 68 Kyoto University of Education, Kyoto, Japan; 69 Department of Physics, Kyushu University, Fukuoka, Japan; 70 Instituto de Física La Plata, Universidad Nacional de La Plata and CONICET, La Plata, Argentina; 71 Physics Department, Lancaster University, Lancaster, UK; 72 INFN Sezione di Lecce; Dipartimento di Matematica e Fisica, Università del Salento, Lecce, Italy; 73 Oliver Lodge Laboratory, University of Liverpool, Liverpool, UK; 74 Department of Physics, Jožef Stefan Institute and University of Ljubljana, Ljubljana, Slovenia; 75 School of Physics and Astronomy, Queen Mary University of London, London, UK; 76 Department of Physics, Royal Holloway University of London, Surrey, UK; 77 Department of Physics and Astronomy, University College London, London, UK; 78 Laboratoire de Physique Nucléaire et de Hautes Energies, UPMC and Université Paris-Diderot and CNRS/IN2P3, Paris, France; 79 Fysiska institutionen, Lunds universitet, Lund, Sweden; 80 Departamento de Fisica Teorica C-15, Universidad Autonoma de Madrid, Madrid, Spain; 81 Institut für Physik, Universität Mainz, Mainz, Germany; 82 School of Physics and Astronomy, University of Manchester, Manchester, UK; 83 CPPM, Aix-Marseille Université and CNRS/IN2P3, Marseille, France; 84 Department of Physics, University of Massachusetts, Amherst, MA USA; 85 Department of Physics, McGill University, Montreal, QC Canada; 86 School of Physics, University of Melbourne, Victoria, Australia; 87 Department of Physics, The University of Michigan, Ann Arbor, MI USA; 88 Department of Physics and Astronomy, Michigan State University, East Lansing, MI USA; 89 INFN Sezione di Milano; Dipartimento di Fisica, Università di Milano, Milano, Italy; 90 B.I. Stepanov Institute of Physics, National Academy of Sciences of Belarus, Minsk, Republic of Belarus; 91 National Scientific and Educational Centre for Particle and High Energy Physics, Minsk, Republic of Belarus; 92 Department of Physics, Massachusetts Institute of Technology, Cambridge, MA USA; 93 Group of Particle Physics, University of Montreal, Montreal, QC Canada; 94 P.N. Lebedev Institute of Physics, Academy of Sciences, Moscow, Russia; 95 Institute for Theoretical and Experimental Physics (ITEP), Moscow, Russia; 96 Moscow Engineering and Physics Institute (MEPhI), Moscow, Russia; 97 Skobeltsyn Institute of Nuclear Physics, Lomonosov Moscow State University, Moscow, Russia; 98 Fakultät für Physik, Ludwig-Maximilians-Universität München, München, Germany; 99 Max-Planck-Institut für Physik (Werner-Heisenberg-Institut), München, Germany; 100 Nagasaki Institute of Applied Science, Nagasaki, Japan; 101 Graduate School of Science and Kobayashi-Maskawa Institute, Nagoya University, Nagoya, Japan; 102 INFN Sezione di Napoli; Dipartimento di Scienze Fisiche, Università di Napoli, Napoli, Italy; 103 Department of Physics and Astronomy, University of New Mexico, Albuquerque, NM USA; 104 Institute for Mathematics, Astrophysics and Particle Physics, Radboud University Nijmegen/Nikhef, Nijmegen, Netherlands; 105 Nikhef National Institute for Subatomic Physics and University of Amsterdam, Amsterdam, Netherlands; 106 Department of Physics, Northern Illinois University, DeKalb, IL USA; 107 Budker Institute of Nuclear Physics, SB RAS, Novosibirsk Russia; 108 Department of Physics, New York University, New York, NY USA; 109 Ohio State University, Columbus, OH USA; 110 Faculty of Science, Okayama University, Okayama, Japan; 111 Homer L. Dodge Department of Physics and Astronomy, University of Oklahoma, Norman, OK USA; 112 Department of Physics, Oklahoma State University, Stillwater, OK USA; 113 Palacký University, RCPTM, Olomouc Czech Republic; 114 Center for High Energy Physics, University of Oregon, Eugene, OR USA; 115 LAL, Université Paris-Sud and CNRS/IN2P3, Orsay, France; 116 Graduate School of Science, Osaka University, Osaka, Japan; 117 Department of Physics, University of Oslo, Oslo, Norway; 118 Department of Physics, Oxford University, Oxford, UK; 119 INFN Sezione di Pavia; Dipartimento di Fisica, Università di Pavia, Pavia, Italy; 120 Department of Physics, University of Pennsylvania, Philadelphia, PA USA; 121 Petersburg Nuclear Physics Institute, Gatchina, Russia; 122 INFN Sezione di Pisa; Dipartimento di Fisica E. Fermi, Università di Pisa, Pisa, Italy; 123 Department of Physics and Astronomy, University of Pittsburgh, Pittsburgh, PA USA; 124 Laboratorio de Instrumentacao e Fisica Experimental de Particulas, LIP, Lisboa, Portugal; Departamento de Fisica Teorica y del Cosmos and CAFPE, Universidad de Granada, Granada, Spain; 125 Institute of Physics, Academy of Sciences of the Czech Republic, Praha, Czech Republic; 126 Faculty of Mathematics and Physics, Charles University in Prague, Praha, Czech Republic; 127 Czech Technical University in Prague, Praha, Czech Republic; 128 State Research Center Institute for High Energy Physics, Protvino, Russia; 129 Particle Physics Department, Rutherford Appleton Laboratory, Didcot, UK; 130 Physics Department, University of Regina, Regina, SK Canada; 131 Ritsumeikan University, Kusatsu, Shiga Japan; 132 INFN Sezione di Roma I; Dipartimento di Fisica, Università La Sapienza, Roma, Italy; 133 INFN Sezione di Roma Tor Vergata; Dipartimento di Fisica, Università di Roma Tor Vergata, Roma, Italy; 134 INFN Sezione di Roma Tre; Dipartimento di Fisica, Università Roma Tre, Roma, Italy; 135 Faculté des Sciences Ain Chock, Réseau Universitaire de Physique des Hautes Energies, Université Hassan II, Casablanca; Centre National de l’Energie des Sciences Techniques Nucleaires, Rabat; Faculté des Sciences Semlalia, Université Cadi Ayyad, LPHEA-Marrakech; Faculté des Sciences, Université Mohamed Premier and LPTPM, Oujda; Faculté des sciences, Université Mohammed V-Agdal, Rabat, Morocco; 136 DSM/IRFU (Institut de Recherches sur les Lois Fondamentales de l’Univers), CEA Saclay (Commissariat a l’Energie Atomique), Gif-sur-Yvette, France; 137 Santa Cruz Institute for Particle Physics, University of California Santa Cruz, Santa Cruz, CA USA; 138 Department of Physics, University of Washington, Seattle, WA USA; 139 Department of Physics and Astronomy, University of Sheffield, Sheffield, UK; 140 Department of Physics, Shinshu University, Nagano, Japan; 141 Fachbereich Physik, Universität Siegen, Siegen, Germany; 142 Department of Physics, Simon Fraser University, Burnaby, BC Canada; 143 SLAC National Accelerator Laboratory, Stanford, CA USA; 144 Faculty of Mathematics, Physics & Informatics, Comenius University, Bratislava; Department of Subnuclear Physics, Institute of Experimental Physics of the Slovak Academy of Sciences, Kosice, Slovak Republic; 145 Department of Physics, University of Johannesburg, Johannesburg; School of Physics, University of the Witwatersrand, Johannesburg, South Africa; 146 Department of Physics, Stockholm University; The Oskar Klein Centre, Stockholm, Sweden; 147 Physics Department, Royal Institute of Technology, Stockholm, Sweden; 148 Departments of Physics & Astronomy and Chemistry, Stony Brook University, Stony Brook, NY USA; 149 Department of Physics and Astronomy, University of Sussex, Brighton, UK; 150 School of Physics, University of Sydney, Sydney, Australia; 151 Institute of Physics, Academia Sinica, Taipei, Taiwan; 152 Department of Physics, Technion: Israel Institute of Technology, Haifa, Israel; 153 Raymond and Beverly Sackler School of Physics and Astronomy, Tel Aviv University, Tel Aviv, Israel; 154 Department of Physics, Aristotle University of Thessaloniki, Thessaloniki, Greece; 155 International Center for Elementary Particle Physics and Department of Physics, The University of Tokyo, Tokyo, Japan; 156 Graduate School of Science and Technology, Tokyo Metropolitan University, Tokyo, Japan; 157 Department of Physics, Tokyo Institute of Technology, Tokyo, Japan; 158 Department of Physics, University of Toronto, Toronto, ON Canada; 159 TRIUMF, Vancouver BC; Department of Physics and Astronomy, York University, Toronto, ON Canada; 160 Faculty of Pure and Applied Sciences, University of Tsukuba, Tsukuba, Japan; 161 Department of Physics and Astronomy, Tufts University, Medford, MA USA; 162 Centro de Investigaciones, Universidad Antonio Narino, Bogota, Colombia; 163 Department of Physics and Astronomy, University of California Irvine, Irvine, CA USA; 164 INFN Gruppo Collegato di Udine; ICTP, Trieste; Dipartimento di Chimica, Fisica e Ambiente, Università di Udine, Udine, Italy; 165 Department of Physics, University of Illinois, Urbana, IL USA; 166 Department of Physics and Astronomy, University of Uppsala, Uppsala, Sweden; 167 Instituto de Física Corpuscular (IFIC) and Departamento de Física Atómica, Molecular y Nuclear and Departamento de Ingeniería Electrónica and Instituto de Microelectrónica de Barcelona (IMB-CNM), University of Valencia and CSIC, Valencia, Spain; 168 Department of Physics, University of British Columbia, Vancouver, BC Canada; 169 Department of Physics and Astronomy, University of Victoria, Victoria, BC Canada; 170 Department of Physics, University of Warwick, Coventry, UK; 171 Waseda University, Tokyo, Japan; 172 Department of Particle Physics, The Weizmann Institute of Science, Rehovot, Israel; 173 Department of Physics, University of Wisconsin, Madison, WI USA; 174 Fakultät für Physik und Astronomie, Julius-Maximilians-Universität, Würzburg, Germany; 175 Fachbereich C Physik, Bergische Universität Wuppertal, Wuppertal, Germany; 176 Department of Physics, Yale University, New Haven, CT USA; 177 Yerevan Physics Institute, Yerevan, Armenia; 178 Centre de Calcul de l’Institut National de Physique Nucléaire et de Physique des Particules (IN2P3), Villeurbanne, France; 179 CERN, 1211 Geneva 23, Switzerland

## Abstract

A measurement is presented of the $\phi \times \mathcal {BR}(\phi \rightarrow K^{+}K^{-})$ production cross section at $\sqrt{s}$ = 7 TeV using $pp$ collision data corresponding to an integrated luminosity of 383 $\mathrm {\upmu b^{-1}}$, collected with the ATLAS experiment at the LHC. Selection of $\phi$(1020) mesons is based on the identification of charged kaons by their energy loss in the pixel detector. The differential cross section is measured as a function of the transverse momentum, $p_{\mathrm {T,\phi }}$, and rapidity, $y_{\phi }$, of the $\phi$(1020) meson in the fiducial region 500 $<p_{\mathrm {T,\phi }}<$ 1200 MeV, $|y_{\phi }|<$ 0.8, kaon $p_{\mathrm {T},K}>$ 230 MeV and kaon momentum $p_{K}<$ 800 MeV. The integrated $\phi (1020)$-meson production cross section in this fiducial range is measured to be $\sigma _{\phi } \times \mathcal {BR}(\phi \rightarrow K^{+}K^{-})$ = 570 $\pm$ 8 (stat) $\pm$ 66 (syst) $\pm$ 20 (lumi) $\mathrm {\upmu b}$.

## Introduction

Perturbative quantum chromodynamics (QCD) successfully describes physics of hadronic interactions at high momentum transfer ($Q^2 \gg 1~\mathrm{GeV}^{2}$), while phenomenological models are needed for soft interactions at lower momentum transfers. An accurate description of these soft interactions is required to model so-called underlying events present in hard scattering events. Measurements of the $\phi$ (1020)-meson probe strangeness production at a soft scale $Q \sim$ 1 GeV, which is sensitive to $s$-quark and low-$x$ ($x \sim 10^{-4}$) gluon densities. The measurement is therefore sensitive to the proton parton distribution function (PDF), which is used by Monte Carlo (MC) generators to describe the longitudinal momentum distributions of the proton’s constituent partons. Production of $\phi$(1020) mesons is also sensitive to fragmentation details and thus $\phi$(1020) measurements can constrain phenomenological soft hadroproduction models.

This paper presents a measurement with the ATLAS detector [1] of the $\phi (1020)$-meson production cross section in $pp$ interactions at $\sqrt{s}=7$ TeV, using the $\phi \rightarrow K^{+}K^{-}$ decay mode. The cross section is not corrected for the branching fraction in the fiducial range. The cross section is measured in bins of transverse momentum, $p_{\mathrm {T,\phi }}$, or of rapidity $|y_{\phi }|$.^1^ The selection of $\phi$(1020)-meson candidates requires the identification of kaons in order to reduce the large combinatorial background from other charged particles. Charged particles are reconstructed with the inner detector, which consists of a silicon pixel detector, a microstrip semiconductor tracker (SCT), and a straw-tube transition radiation tracker (TRT). The inner detector barrel (end-cap) parts consist of 3 (2 $\times$ 3) pixel layers, 4 (2 $\times$ 9) layers of double-sided silicon strip modules, and 73 (2 $\times$ 160) layers of TRT straws. A track traversing the barrel typically has 11 silicon hits (3 pixel clusters, and 8 strip clusters), and more than 30 straw-tube hits. The whole inner detector is immersed in a 2 T axial magnetic field. The specific energy loss of charged particles in the pixel detector is used to identify low-momentum pions, kaons and protons [2].

To avoid model-dependent extrapolations outside the detector acceptance, the cross section is measured in the fiducial region, defined as 500 $<p_{\mathrm {T,\phi }}<$ 1200 MeV, $|y_{\phi }|<$ 0.8, kaon transverse momentum $p_{\mathrm {T},K}>$ 230 MeV and kaon momentum $p_{K}<$ 800 MeV. In the region 0.8 $<|y_{\phi }|<$ 1.0, $\phi$(1020) decays would only be accepted up to $p_{\mathrm {T,\phi }}\sim$ 700 MeV, because the requirement of $p_{K}<$ 800 MeV has a lower efficiency at higher rapidity. The fiducial range is limited to the region where the differential cross section can be measured and where correcting for the losses due to the requirements on kaon momentum is reliable. The measurement is corrected for detector effects and can be compared directly with MC generators at particle level.

Many measurements of the $\phi$(1020) production cross section have been performed at different centre-of-mass energies, using different decay modes and in different rapidity ranges. Among these are a study at $\sqrt{s}=7$ TeV by ALICE [3] in a similar rapidity region and another by LHCb [4] in the forward rapidity region. The $\phi$(1020) production cross section presented in this paper is compared to the measurement by ALICE and to MC predictions.

## Data set and event selection

A data sample with an integrated luminosity of 383 $\mathrm {\upmu b^{-1}}$ from $pp$ collision data taken in April 2010 at $\sqrt{s}=7$ TeV is used. The contribution of pile-up, i.e. multiple collisions per bunch crossing, is negligible for this data sample, with a peak luminosity of $1.8 \times 10^{28} \mathrm {\,\,cm^{-2}\,\,s^{-1}}$. The luminosity is measured in dedicated van der Meer scans with an estimated uncertainty of 3.5 % [5]. The data sample was selected with the minimum bias trigger scintillators (MBTS) [6] to minimize any possible bias in the measured cross section. The MBTS are mounted at each end of the tracking detector in front of the liquid-argon endcap-calorimeter cryostats at $z$ = $\pm$ 3.56 m and were configured to require one hit above threshold from either side of the detector. This trigger is shown to be highly efficient in selecting inelastic $pp$ collisions [6]. Tracks are fitted with a kaon-mass assumption to account for energy losses in the detector material. Events are required to contain at least two tracks with $p_\mathrm{T} >$ 150 MeV and to have a primary vertex (PV, defined as the vertex in the event with the largest $\Sigma p_\mathrm{T}$ over all reconstructed tracks associated to the vertex) [7] reconstructed using the beam spot information [6].

MC simulations are used to correct the data for detector effects and to compare with the fully corrected data. The MC generators used are PYTHIA6 [8], PYTHIA8 [9], Herwig++ [10] and EPOS [11, 12]. Different versions of the same MC generator, that differ in sets of tunable parameters used in modeling the soft component of proton-proton interactions, are called tunes. Both PYTHIA6 and PYTHIA8 are general purpose generators which implement the Lund string hadronisation model [13] and describe non-diffractive interactions (including Multiple Parton Interactions, MPI) via lowest-order perturbative QCD, with phenomenological regularisation of the divergence of the cross section as $p_\mathrm{T} \,{\rightarrow }\, 0$. Diffractive processes are included which involve the exchange of a colour singlet. Both inelastic non-diffractive and diffractive processes are mixed in accordance with the generator cross sections. The PYTHIA tunes considered are MC09 [14] with PYTHIA6 version 6.421, DW [15] and Perugia0 [16] with PYTHIA6 version 6.423, and two A2 tunes with PYTHIA8 version 8.153, i.e. with the MSTW2008LO [17, 18] and CTEQ6L1 [19] PDF sets. The MC09 and Perugia0 tunes use a $p_\mathrm{T}$-ordered parton shower model with MPI and the initial-state shower interleaved in a common sequence of decreasing $p_\mathrm{T}$. For the PYTHIA8 A2 tunes, the final-state showers are also interleaved in this way. The DW tune utilises the older virtuality-ordered parton shower which is not interleaved with MPI.

Herwig++ version 2.5.1 is used with the UE7-2 [20] tune. Herwig++ is also a general purpose generator but differs from PYTHIA in that it uses a cluster hadronisation model [21] and an angular-ordered parton shower. Herwig++ contains a tunable eikonalised MPI model which assumes independence between separate scatters in the event. In order to simulate inelastic minimum bias events the following mechanism is used. For a fixed impact parameter, Poisson distributions are sampled to provide the number of soft and perturbatively-treated semi-hard scatters to simulate per event.

EPOS 1.99 v2965 is used with the EPOS-LHC [22] tune. EPOS contains a parametrised approximation of the hydrodynamic evolution of initial states using a parton based Gribov-Regge [23] theory which has been tuned to LHC data.

The ATLAS detector is simulated [24] using GEANT4 [25]. The reconstruction of $K^{\pm }$ tracks from $\phi \rightarrow K^{+}K^{-}$ decays generated by PYTHIA6 MC09 is used for the calculation of the tracking efficiency. A consistency test of the full $\phi$(1020)-meson reconstruction is performed with PYTHIA6 MC09 and Herwig++ UE7-2.

As the $\phi$(1020) meson has no measurable decay length, only tracks originating from the PV are used. Each track must pass the following requirements: more than one pixel cluster and more than one SCT hit; $p_\mathrm{T}>$ 230 MeV; $p<$ 800 MeV and $|\eta |<$ 2.0. The condition $p_\mathrm{T}>$ 230 MeV is adopted since the tracking efficiency for kaon tracks with $p_{\mathrm {T},K}<$ 230 MeV and central $|\eta |$ is close to zero. Kaons produced with such low momenta effectively deposit all their energy in the detector and support materials before reaching the SCT. The cut on track momentum of $p<$ 800 MeV is dictated by particle identification requirements and is explained in the next section.

## Particle identification

**Fig. 1:**
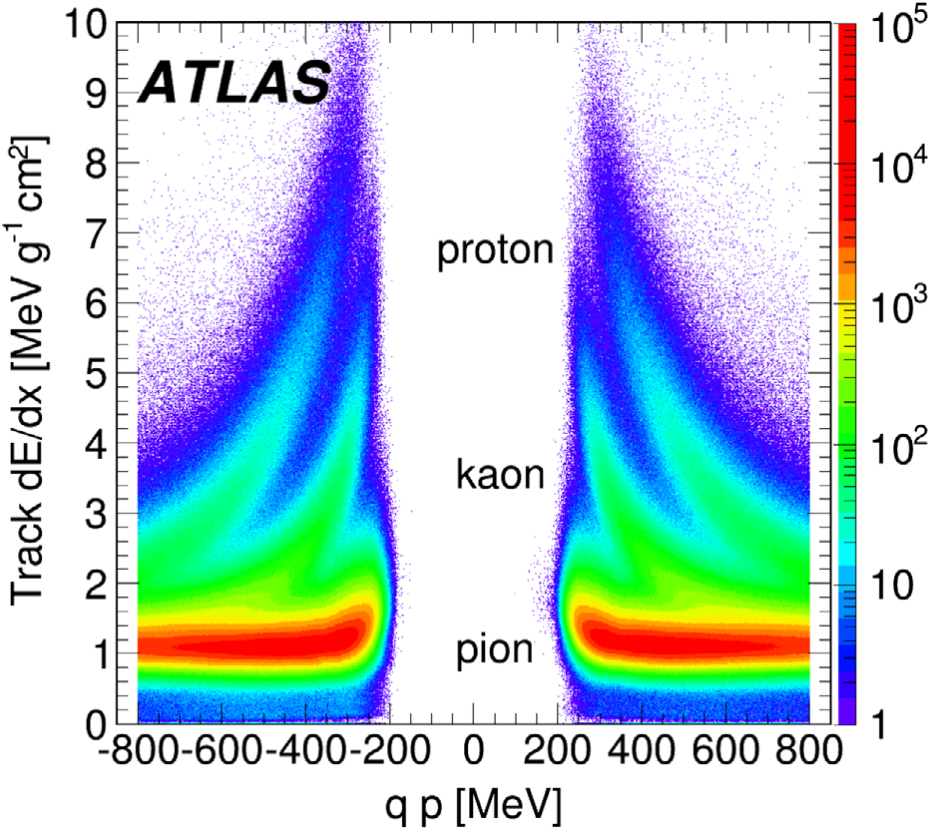
The truncated mean (see text for detailed explanation) for the energy loss per track as a function of signed momentum for tracks accepted in the analysis. The bands corresponding to the energy lost by pions, kaons and protons are labelled

Every pair of oppositely charged tracks passing the tracking cuts is examined. The identification of a pair of tracks candidate for a $\phi \rightarrow K^{+}K^{-}$ decay requires a particle identification (PID) step to remove the large combinatorial background from pairs containing one or two charged particles that are not kaons. Discrimination between background (consisting mostly of pions) and kaons is achieved using energy loss in the pixel detector. The mean energy deposited by a charged particle is described by the Bethe–Bloch formula as a function of the particle’s velocity [26]. For momenta larger than 1 GeV, the energy lost by the particles starts to be dominated by relativistic effects and can no longer be used for particle identification. The mean energy loss per unit length is estimated as the energy deposited by a particle in the traversed layers of the pixel detector divided by the local thickness traversed in the detector material. The energy deposited is calculated after removing the pixel cluster with the largest charge for particles with three or four associated pixel clusters or after removing the two clusters with the largest charge for particles with more than four pixel clusters. The track $\mathrm {d}E/\mathrm {d}x$ is calculated using a truncated mean of the $\mathrm {d}E/\mathrm {d}x$ values of the individual pixel clusters as this gives a better resolution than the simple mean. The expected energy loss for a kaon with $p_{K}=$ 500 MeV is 2.4 MeV $\mathrm {g^{-1}}$ $\mathrm {cm^{2}}$. For a pion with the same momentum, an energy loss of 1.2 MeV $\mathrm {g^{-1}}$ $\mathrm {cm^{2}}$ is expected. The average energy loss per track as a function of signed momentum, $qp$, where $q$ is the particle charge, is shown in Fig. 1; bands indicating pions, kaons and protons are clearly visible.

**Fig. 2:**
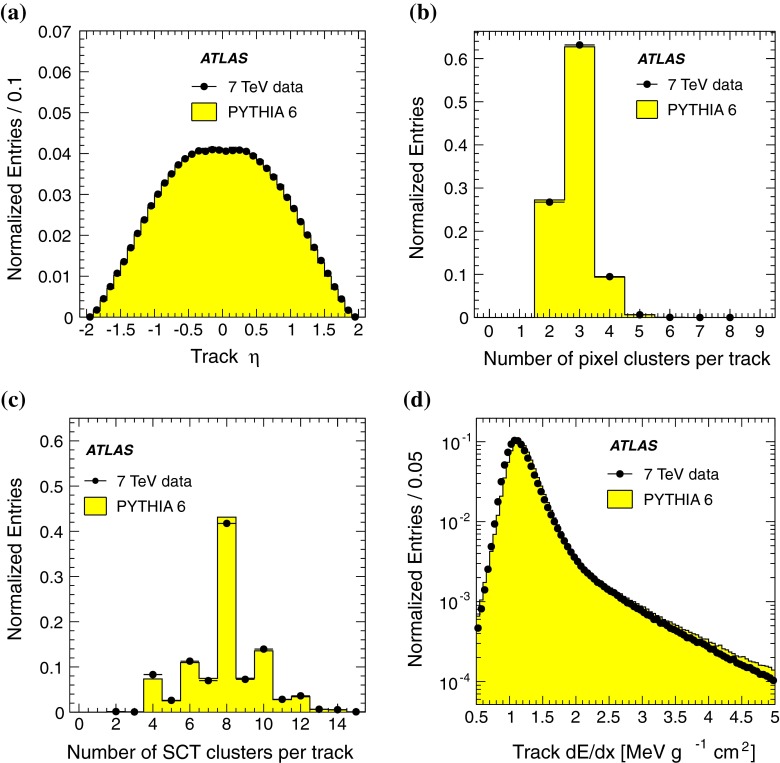
Comparison between data (*black dots*) and MC simulation (*histogram*) for **a** track $\eta$, **b** number of pixel clusters assigned to the track, **c** number of SCT clusters assigned to the track and **d** the average track energy loss (see text). Statistical uncertainties are smaller than the marker size

A comparison between data and MC prediction of track $\eta$, of the number of hits in the pixel and SCT detectors associated with tracks (with a requirement of at least two pixel clusters and two SCT hits) and of average energy loss per track is presented in Fig. 2. The distributions agree well, demonstrating a good understanding of track simulation and reconstruction in the inner detector. The slight disagreement in Fig. 2d, where the location of the peak of the average energy loss is overestimated by $\sim$0.05 MeV $\mathrm {g^{-1}}$ $\mathrm {cm^{2}}$ in the MC simulation, is due to the relative abundances of different particle species being slightly different for data and simulation.

The most probable value of the specific energy loss for a pion, kaon or proton hypothesis is parameterized as a function of the charged particle’s Lorentz factor $\beta \gamma$. The measured energy loss is used to calculate the probability $P_{\mathrm {particle}}$ of compatibility with a given hypothesis [2]. Kaon candidates are required to satisfy $P_{\mathrm {pion}}< 0.1$ and $P_{\mathrm {kaon}}> 0.84$ conditions. The candidate $\phi$(1020) decays are searched for by selecting the oppositely charged track pairs for which both tracks pass the tracking and PID requirements defined above and combine to an invariant mass in the range 1000 $<$ $m(K^+K^-)$ $<$ 1060 MeV.

## Determination of the cross section

The fiducial region is divided into eight bins in $|y_{\phi }|$ and ten bins in $p_{\mathrm {T,\phi }}$ with bin widths of 0.1 and 70 MeV, respectively. Unless specifically stated, the cross section is not corrected for the branching fraction of $\phi$(1020)-meson decays to kaons.

Each $\phi$(1020) candidate is assigned a weight to correct for experimental losses. Firstly, a weight is given for trigger and vertex reconstruction efficiencies [6], which have both been measured in data to rapidly increase to 100 % for events with four or more tracks. The trigger and vertex reconstruction efficiencies were found to have a negligible effect on this analysis and were applied on an event-by-event basis. Secondly, a weight is given for track reconstruction and kaon identification efficiencies on a track-by-track basis. These efficiencies are calculated separately for tracks from positively and negatively charged particles, because fewer pixel clusters are expected on the tracks of low-momentum negatively charged particles, which may pass in between two pixel modules due to the tiling and tilt of the modules. The average number of pixel clusters on tracks which pass the selection detailed in Sect. 2 is $2.96 \pm 0.01$ per positively charged particle and $2.79 \pm 0.01$ per negatively charged particle. Finally, a weight is given on a track-by-track basis to correct for the fraction of selected tracks passing the kinematic selection for which the corresponding generated kaon is outside the kinematic range. Following the determination of the weight of each of the candidate $\phi$(1020), the efficiency-corrected number of reconstructed candidates is determined with a fit to the invariant mass distribution.

The calculation of track reconstruction efficiency, kaon identification efficiency and the subsequent signal yield extraction are explained in the next sections.

### Track reconstruction efficiency

The track reconstruction efficiency, $\epsilon _{\mathrm {rec}}$, is based on MC ‘truth-matching’, where generated particles are matched to reconstructed tracks. The simulation-based method is based on a matching probability evaluated using the number of common hits between particles at generator level and the reconstructed tracks, and is described in Ref. [6]. The average tracking efficiency for the two tracks of a $\phi \rightarrow K^{+}K^{-}$ decay is about 40 % for the lower $p_{\mathrm {T,\phi }}$ bins and increases to 65 % in the highest $p_{\mathrm {T,\phi }}$ bin. It is $\sim$ 50 % for all bins in rapidity.

Only to estimate the quality of the MC description of $\epsilon _{\mathrm {rec}}$ in data, the number of tracks passing all cuts in bins of pseudorapidity is divided by the number of tracks passing the cuts with one cut loosened. This efficiency is referred to as the relative efficiency $\epsilon _{\mathrm {rel}}$. The behavior of $\epsilon _{\mathrm {rel}}$ with one fewer pixel cluster or one fewer SCT hit required per track and a lower momentum cut is compared between simulation and data and found to be consistent within 0.5 %. The systematic uncertainty inferred is 0.7 % per track pair.

The dominant source of uncertainty is due to uncertainty in the MC material description, denoted as $\epsilon _{\mathrm {rec}}$(material). It is described in Ref. [6] and is given in bins of track $\eta$ and $p_\mathrm{T}$. The material uncertainty, expressed as a fraction of the corresponding tracking efficiency, is 2–3 % for most tracks accepted in this analysis. To evaluate the impact of this uncertainty, the yield is extracted with the nominal tracking efficiency, and with the nominal tracking efficiency varied up and down by this uncertainty. The systematic uncertainty arising from $\epsilon _{\mathrm {rec}}$(material) is accounted for per bin in $p_{\mathrm {T,\phi }}$ or $|y_{\phi }|$ and is 5 % per track pair.

The number of reconstructed decays is corrected for the fraction of selected tracks passing the kinematic selection for which the corresponding primary particle is outside the kinematic range. The distributions are subsequently corrected using a MC derived factor to account for the migration of reconstructed $\phi$(1020)-meson candidates into the fiducial volume. The systematic uncertainty arising from this migration correction is evaluated by re-calculating the migration correction after re-weighting the kaon momentum spectrum at particle-level to get a good description of the data at detector level. The variation of the extracted yield using the default and re-weighted migration correction is assigned as a systematic uncertainty and is below 1 %.

The statistical uncertainty on the tracking efficiency, $\epsilon _{\mathrm {rec}}$(stat), is in the range 1–5 % and is propagated as a systematic uncertainty on the cross section. The total systematic uncertainty in the tracking efficiency determination is obtained by adding the previously mentioned components in quadrature and is summarized in Tables 1 and 2 as a function of $p_{\mathrm {T,\phi }}$ and $|y_{\phi }|$, respectively.

**Table 1:** The fitted number of $\phi$(1020) candidates (Signal), the differential production cross section $\mathrm {d}\sigma /\mathrm {d} p_\mathrm{T}$ ($\upmu$b/MeV) of $\phi \rightarrow K^{+} K^{-}$ and its statistical uncertainty in bins of $p_{\mathrm {T,\phi }}$ with 500 $<p_{\mathrm {T,\phi }}<$ 1200 MeV, $|y_{\phi }|<$ 0.8, $p_{\mathrm {T},K}>$ 230 MeV and $p_{K}<$ 800 MeV and the systematic uncertainties due to track reconstruction efficiency ($\epsilon _{\mathrm {rec}}$), kaon identification ($\epsilon _{\mathrm {pid}}$) and fitting procedure. The uncertainty on the luminosity is 3.5 %

Bin (MeV)	Signal (in units of $10^{4}$)	$\mathrm {d}\sigma /\mathrm {d}p\hbox {T}$ ($\upmu$b/MeV)	Systematic uncertainty ($\upmu$b/MeV)		
			$\epsilon _{\mathrm {rec}}$	$\epsilon _{\mathrm {pid}}$	Fitting
$500<p_{\mathrm {T,\phi }}\le 570$	1.22 $\pm$ 0.07	0.44 $\pm$ 0.03	${} \pm 0.03$	${} \pm 0.03$	${} \pm 0.03$
$570< p_{\mathrm {T,\phi }}\le 640$	$2.34 \pm 0.09$	$0.87 \pm 0.04$	${} \pm 0.06$	${} \pm 0.05$	${} \pm 0.05$
$640<p_{\mathrm {T,\phi }}\le 710$	$2.71 \pm 0.10$	$1.01 \pm 0.04$	${} \pm 0.06$	${} \pm 0.06$	${} \pm 0.06$
$710<p_{\mathrm {T,\phi }}\le 780$	$3.19 \pm 0.11$	$1.19 \pm 0.04$	${} \pm 0.07$	${} \pm 0.09$	${} \pm 0.07$
$780<p_{\mathrm {T,\phi }}\le 850$	$3.16 \pm 0.11$	$1.18 \pm 0.04$	${} \pm 0.06$	${} \pm 0.10$	${} \pm 0.07$
$850<p_{\mathrm {T,\phi }}\le 920$	$2.85 \pm 0.10$	$1.05 \pm 0.04$	${} \pm 0.05$	${} \pm 0.09$	${} \pm 0.06$
$920<p_{\mathrm {T,\phi }}\le 990$	$2.15 \pm 0.09$	$0.79 \pm 0.04$	${} \pm 0.03$	${} \pm 0.08$	${} \pm 0.06$
$990<p_{\mathrm {T,\phi }}\le 1060$	$1.81 \pm 0.07$	$0.67 \pm 0.04$	${} \pm 0.03$	${} \pm 0.07$	${} \pm 0.05$
$1060<p_{\mathrm {T,\phi }}\le 1130$	$1.30 \pm 0.06$	$0.48 \pm 0.04$	${} \pm 0.02$	${} \pm 0.05$	${} \pm 0.03$
$1130<p_{\mathrm {T,\phi }}\le 1200$	$1.23 \pm 0.08$	$0.46 \pm 0.04$	${} \pm 0.02$	${} \pm 0.06$	${} \pm 0.03$

**Table 2:** The fitted number of $\phi$(1020) candidates (Signal), the differential production cross section $\mathrm {d}\sigma /\mathrm {d}|y|$ (mb) of $\phi \rightarrow K^{+} K^{-}$ and its statistical uncertainty in bins of $|y_{\phi }|$ with 500 $<p_{\mathrm {T,\phi }}<$ 1200 MeV, $|y_{\phi }|<$ 0.8, $p_{\mathrm {T},K}>$ 230 MeV and $p_{K}<$ 800 MeV and the systematic uncertainties due to track reconstruction efficiency ($\epsilon _{\mathrm {rec}}$), kaon identification ($\epsilon _{\mathrm {pid}}$) and fitting procedure. The uncertainty on the luminosity is 3.5 %

Bin	Signal (in units of $10^{4}$)	$\mathrm {d}\sigma /\mathrm {d}|y|$ (mb)	Systematic uncertainty (mb)		
			$\epsilon _{\mathrm {rec}}$	$\epsilon _{\mathrm {pid}}$	Fitting
$0.0<|y_{\mathrm {\phi }}| \le 0.1$	$3.44 \pm 0.10$	$0.90 \pm 0.03$	${} \pm 0.04$	${} \pm 0.06$	${} \pm 0.05$
$0.1<|y_{\mathrm {\phi }}| \le 0.2$	$3.39 \pm 0.10$	$0.88 \pm 0.03$	${} \pm 0.04$	${} \pm 0.07$	${} \pm 0.05$
$0.2<|y_{\mathrm {\phi }}| \le 0.3$	$3.22 \pm 0.09$	$0.84 \pm 0.03$	${} \pm 0.04$	${} \pm 0.06$	${} \pm 0.05$
$0.3<|y_{\mathrm {\phi }}| \le 0.4$	$3.18 \pm 0.09$	$0.82 \pm 0.03$	${} \pm 0.04$	${} \pm 0.06$	${} \pm 0.05$
$0.4<|y_{\mathrm {\phi }}| \le 0.5$	$3.36 \pm 0.11$	$0.88 \pm 0.03$	${} \pm 0.05$	${} \pm 0.08$	${} \pm 0.05$
$0.5<|y_{\mathrm {\phi }}| \le 0.6$	$2.53 \pm 0.12$	$0.66 \pm 0.03$	${} \pm 0.04$	${} \pm 0.06$	${} \pm 0.04$
$0.6<|y_{\mathrm {\phi }}| \le 0.7$	$2.01 \pm 0.11$	$0.51 \pm 0.02$	${} \pm 0.03$	${} \pm 0.05$	${} \pm 0.04$
$0.7<|y_{\mathrm {\phi }}| \le 0.8$	$1.18 \pm 0.07$	$0.30 \pm 0.02$	${} \pm 0.02$	${} \pm 0.04$	${} \pm 0.02$

### Particle identification efficiency

The particle identification efficiency, $\epsilon _{\mathrm {pid}}$, is extracted from simulation as a function of both $p_{K}$ and $\eta$. The data sample is not large enough to determine the PID efficiency with a purely data driven technique in bins of $p_{K}$ and $\eta$. Therefore a data-driven tag-and-probe technique is used to determine the PID in bins of $p_{K}$ and this is used to rescale the Monte Carlo estimates of the PID efficiency. The data sample is split up into five bins of $p_{K}$ and the efficiency is measured as the fraction $N_{\mathrm {probe}}/N_{\mathrm {tag}}$, where $N_{\mathrm {probe}}$ is the number of candidates for which both kaons pass the PID requirement of $P_{\mathrm {pion}}<$ 0.1 and $P_{\mathrm {kaon}}>$ 0.84, and $N_{\mathrm {tag}}$ is the number of candidates for which at least the $K^{+}$ or the $K^{-}$ passes. To measure the signal yields $N_{\mathrm {tag}}$ and $N_{\mathrm {probe}}$, the invariant mass distribution in each bin of $p_{K}$ is fitted with a probability density function (p.d.f.) that describes the signal and background contributions separately and which is detailed in Sect. 4.3. A final efficiency correction factor is defined by multiplying the two-dimensional efficiency from MC simulation by the ratio of data to MC tag-and-probe efficiencies, which is close to unity for $p_{K}<$ 500 MeV, but decreases to a factor of slightly more than 0.3 for 700 $<p_{K}<$ 800 MeV. The decreasing efficiency is due to the decreasing discrimination power using energy loss with increasing momentum, seen in Fig. 1, where from $p_{K}\sim$ 600 MeV the bands start to overlap.

The tag-and-probe method is validated using MC simulation by ascertaining that the $\epsilon _{\mathrm {pid}}$ values obtained using MC truth-matching and the tag-and-probe method in bins of $p_{\mathrm {T,\phi }}$ and $|y_{\phi }|$ agree within MC statistical uncertainties. The particle identification efficiency decreases with increasing average kaon momentum from $\sim$90 % for 230 $< p_{K}\le$ 400 MeV to $\sim$10 % for 700 $<p_{K}<$ 800 MeV.

The systematic uncertainty due to $\epsilon _{\mathrm {pid}}$ is evaluated by fixing the background shape parameters in the tag sample to the values given by the fit to the same-sign background distribution (a maximum uncertainty of 10 %) and by adding the same-sign background samples to the fitted data sets for the tag-and-probe validation in PYTHIA6 to vary the signal to background ratio (a maximum uncertainty of 6 %). Possible dependence of the cross section on the choice of $P_{\mathrm {kaon}}$ requirement is tested by varying the requirement by 10 % and is found to be well within the uncertainty due to fixing the background shape parameters. The statistical uncertainty on $\epsilon _{\mathrm {pid}}$ is calculated using a binomial probability distribution, which leads to a relative uncertainty on $\epsilon _{\mathrm {pid}}$ of at most 5 %, denoted by $\epsilon _{\mathrm {pid}}$(stat). These uncertainties (evaluated per bin in $p_{\mathrm {T,\phi }}$ or $|y_{\phi }|$) are added in quadrature and are included as systematic uncertainties on the cross section as summarized in Tables 1 and 2.

### Signal extraction

To extract the signal yields, a binned $\chi ^2$ fit to the invariant mass spectrum is performed in each region of phase space after applying corrections for the selection efficiencies to the tracks. The signal shape is described by a relativistic Breit–Wigner,

1 $$\begin{aligned} f_{\mathrm {RBW}}(m;m_{0},\Gamma_{0}) = \frac{m^{2}}{(m^{2}-m_{0}^{2})^{2} + m_{0}^{2} \Gamma^{2}(m)}, \end{aligned}$$

where the mass-dependent width is given by

2 $$\begin{aligned} \Gamma (m) = \Gamma _{0} \left[ \frac{m^{2}-4m_{K}^{2}}{m_{0}^{2}-4m_{K}^{2}} \right] ^{3/2}. \end{aligned}$$

In Eq. (1), $m_{0}$ is fixed to the $\phi (1020)$-meson mass of 1019.45 MeV [27], $\Gamma _{0}$ to the natural width of 4.26 MeV [27], and $m_{K}$ in Eq. (2) is the charged kaon mass [27].

The signal shape is convoluted with a Gaussian resolution function, with the mean and standard deviation left free in the fit. The mean of the Gaussian is interpreted as the actual value of the $\phi$(1020) mass, while its standard deviation corresponds to the experimental resolution. The values obtained from the fits are in the range $\sigma _{\mathrm {exp}} =$1.0–2.5 MeV.

This signal description is added to an empirical background description,

3 $$\begin{aligned} f_{\mathrm {BKG}}(m)&= (1- e^{(2m_{K}-m)/C}) \cdot \left( \frac{m}{2m_{K}}\right) ^{A} \nonumber \\&\quad +\,B\left( \frac{m}{2m_{K}}-1\right) , \end{aligned}$$

where $A$, $B$ and $C$ determine the background shape. Initial values for $A$, $B$ and $C$ are found by fitting the background p.d.f. to a sample of events with two kaons of the same charge. This same-sign sample contains the same sources of combinatorial background as the nominal selection but no true $\phi$(1020) mesons, and so it provides a good initial description of the background shape. It was checked that the background model provides stable fitting results in all bins in $p_{\mathrm {T,\phi }}$ and $|y_{\phi }|$ for the same-sign sample.

**Fig. 3:**
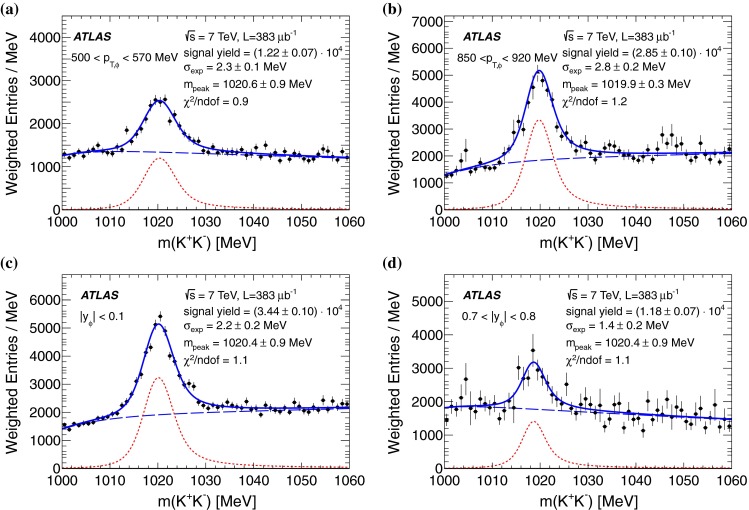
Examples of invariant $K^{+}K^{-}$ mass distributions in the data (*dots*) compared to results of the fits (*solid lines*), as described in the text, for **a** the lowest $p_{\mathrm {T,\phi }}$ bin, **b** one of the middle $p_{\mathrm {T,\phi }}$ bins, **c** the most central $|y_{\phi }|$ bin and **d** most forward $|y_{\phi }|$ bin. The *dashed curves* show the background contribution and the *dotted red curves* demonstrates the signal contributions, with paremeters listed in the legend

Fits of the invariant mass of $K^{+}K^{-}$ pairs are shown in Fig. 3 for four regions. It was found that the maximum of the signal peak, $m_{\mathrm {peak}}$, is shifted upwards by almost 1 MeV for the lowest $p_{\mathrm {T,\phi }}$ bin. This is covered by the uncertainty on the momentum scale for the low-momentum tracks.

Three tests are conducted to estimate the systematic uncertainty on the extracted signal yield due to uncertainty on the signal, background shape and detector resolution. Firstly, the signal is extracted using a non-relativistic Breit–Wigner lineshape convolved with a Gaussian to describe the signal shape. This leads to a conservative estimate of the uncertainties in the extracted signal of 5–6 %, which are evaluated bin-by-bin in $p_{\mathrm {T,\phi }}$ and $|y_{\phi }|$. Secondly, the extracted yield changes by at most 2 % if the signal shape is convolved with a Crystal Ball [28] resolution function, rather than a Gaussian. Thirdly, the extracted yields vary by at most 3 % if the background p.d.f. is fitted to the sample of same-sign pairs of tracks in each bin and the shape is fixed to the result of this fit. Adding the relative changes in the yield in quadrature, a conservative estimate of 6–7 % is assigned to the systematic uncertainty and summarized in Tables 1 and 2.

The cross section $\sigma ^{ i }_{\mathrm {bin}}$ in bin $i$ is determined by

4 $$\begin{aligned} \sigma ^{ i }_{\mathrm {bin}} = \frac{N_{ i } }{\mathcal {L}}, \end{aligned}$$

where $\mathcal {L}$ is the integrated luminosity and $N_{ i }$ is the number of efficiency-corrected reconstructed $\phi \rightarrow K^{+}K^{-}$ candidates in bin $i$.

## Results

**Fig. 4:**
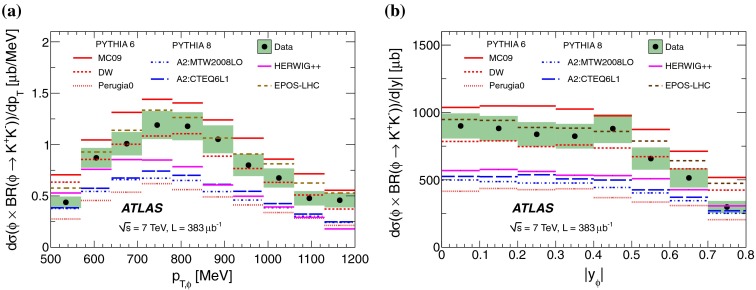
The $\phi \mathrm {(1020)} \times \mathcal {BR}(\phi \rightarrow K^{+}K^{-})$ cross section in the fiducial region, with 500 $< p_{\mathrm {T,\phi }}<$ 1200 MeV, $|y_{\phi }|<$ 0.8, $p_{\mathrm {T},K}>$ 230 MeV and kaon momentum $p_{K}<$ 800 MeV, as a function of $p_{\mathrm {T,\phi }}$ (*left*) and $|y_{\phi }|$ (*right*). The *error bars* represent the statistical uncertainty and the *green boxes* represent the quadratic sum of the statistical and systematic uncertainties. The 3.5 % uncertainty on the luminosity is not included. The data are compared to various MC expectations as described in the legends

The differential $\phi \times \mathcal {BR}(\phi \rightarrow K^{+}K^{-})$ cross section in the fiducial region 500 $< p_{\mathrm {T,\phi }}<$ 1200 MeV, $|y_{\phi }|<$ 0.8, kaon transverse momentum $p_{\mathrm {T},K}>$ 230 MeV and kaon momentum $p_{K}<$ 800 MeV is shown in Fig. 4 a) as a function of $p_{\mathrm {T,\phi }}$ and in Fig. 4 b) as a function of $|y_{\phi }|$ and compared to simulation. Tables 1 and 2 give the differential cross sections and the relevant systematic uncertainties. The total statistical uncertainty ranges from 3 to 8 % and the total systematic uncertainty is 8–12 %. The uncertainty on the luminosity is 3.5 % [5] for all bins. The integrated cross section is calculated as the sum of the differential cross sections as a function of $p_{\mathrm {T,\phi }}$. This determination is less sensitive to mismodelling of the $p_{\mathrm {T,\phi }}$ distribution than a determination based on the sum of the differential cross sections as a function of $|y_{\phi }|$ and is measured to be $\sigma _{\phi } \times \mathcal {BR}(\phi \rightarrow K^{+}K^{-})$ = 570 $\pm$ 8 (stat) $\pm$ 68 (syst) $\pm$ 20 (lumi) $\mathrm {\upmu b}$.

The fiducial cross section increases as a function of $p_{\mathrm {T,\phi }}$ in the range 500–700 MeV, reaches a maximum at $p_{\mathrm {T,\phi }}\sim$ 750 MeV and decreases for $p_{\mathrm {T,\phi }}\ge$ 850 MeV. The increase in the number of measured decays as $p_{\mathrm {T,\phi }}$ rises to 700 MeV is due to the cut on kaon transverse momentum $p_{\mathrm {T},K}>$ 230 MeV, along with the increasing phase space for $\phi$(1020) production. The fiducial cross section is seen to decrease from $|y_{\phi }|\ge$ 0.5. This is due to the $p_{K}<$ 800 MeV requirement for efficient PID which excludes an increasing fraction of kaons as the rapidity increases. The region $|y_{\phi }|< 0.8$ is well within the rapidity plateau at LHC energies, therefore the differential cross section for $\phi$(1020) is expected to be flat as a function of $|y_{\phi }|$ in the measured range of $|y_{\phi }|$.

The cross section is best described by the PYTHIA 6 tune DW and by the EPOS-LHC tune. These provide a good description for the $p_{\mathrm {T,\phi }}$ and $|y_{\phi }|$ dependencies as well as for the total yield. The PYTHIA6 MC09 tune slightly overestimates the data in the fiducial region. The PYTHIA6 Perugia0 tune underestimates the cross section by around a factor of two compared to the data in the whole fiducial volume. The two PYTHIA8 A2 tunes, based on different PDFs, show similar predictions for the cross section, which are also about a factor of two too small. Herwig++ provides a good description for the cross section for $p_{\mathrm {T,\phi }}<700$ MeV and for $|y_{\phi }|>0.6$, but exhibits an overly steeply falling $p_{\mathrm {T,\phi }}$ dependence, such that the cross section is underestimated for $p_{\mathrm {T,\phi }}> 700$ MeV and in the mid-rapidity range $|y_{\phi }|<0.6$.

## Extrapolated cross section

The kaon momenta requirements arising from tracking and PID cuts ($p_{\mathrm {T},K}>$ 230 MeV and $p_{K}<$ 800 MeV) reject a significant number of $\phi \rightarrow K^{+} K^{-}$ candidates. In order to allow comparison with other measurements, the cross section in the fiducial region is extrapolated to a cross section in the kinematic region 500 $<p_{\mathrm {T,\phi }}<$ 1200 MeV and central rapidity $|y_{\phi }|<$ 0.5, using MC particle level information. The variation of the expected correction between the different generators considered is 10 % and is included as an additional systematic uncertainty on the extrapolated result. A correction for the branching fraction is also applied. The systematic uncertainty on the branching fraction is 1 % [27]. The extrapolation is done with PYTHIA6, because the cross section’s dependence on $p_{\mathrm {T,\phi }}$ within the fiducial region is well described by this generator, as shown in Fig. 4. The extrapolation is restricted to $|y_{\phi }|<$ 0.5, where the fiducial acceptance is large, over 70 %. The extrapolation factor is 2.78 for the lowest $p_{\mathrm {T,\phi }}$ bin, then decreases to 1.08 at $p_{\mathrm {T,\phi }}\sim$ 900 MeV and becomes 1.21 in highest $p_{\mathrm {T,\phi }}$ bin.

**Fig. 5:**
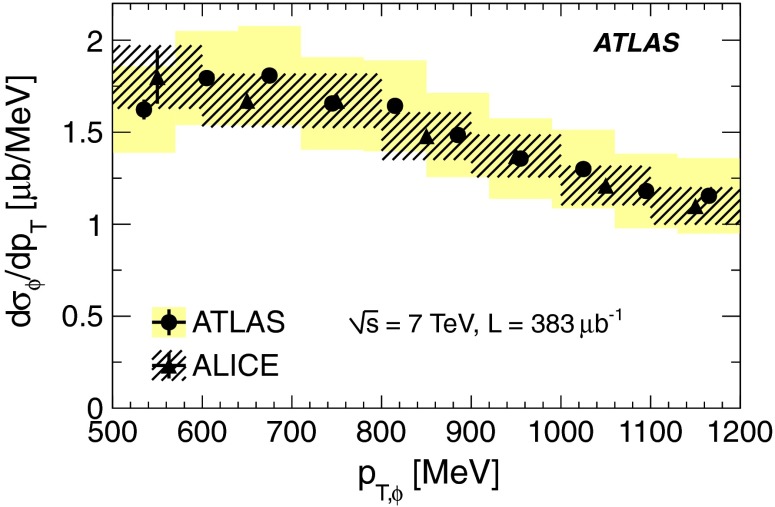
The $\phi$(1020)-meson cross section as a function of $p_{\mathrm {T,\phi }}$, extrapolated using PYTHIA6 to the kinematic region with 500 $< p_{\mathrm {T,\phi }}<$ 1200 MeV and $|y_{\phi }|<$ 0.5, is compared to the measurement by the ALICE Collaboration [3]. The *error bars* represent the statistical uncertainty and the *boxes* represent the quadratic sum of the statistical and systematic uncertainties. The 3.5 % uncertainty on the luminosity is not included

The extrapolated cross section is compared to the measurement by the ALICE Collaboration of the $\phi$(1020) production cross section as described in Ref. [3]. A comparison between the cross section measurements is shown in Fig. 5. The measurements as a function of $p_{\mathrm {T,\phi }}$ are in agreement to within 10 % in the first two bins and to within 3 % in the other bins, which is well within the systematic uncertainties.

## Summary

This paper presents a measurement of the differential production cross section of the $\phi$(1020) meson using the $K^{+}K^{-}$ decay mode and 383 $\mathrm {\upmu b ^{-1}}$ of 7 TeV $pp$ collision data collected with the ATLAS experiment at the LHC. To avoid model-dependent extrapolations outside the detector acceptance, the cross section is measured in a fiducial region, with 500 $< p_{\mathrm {T,\phi }}<$ 1200 MeV, $|y_{\phi }|<$ 0.8, kaon $p_{\mathrm {T},K}>$ 230 MeV and kaon momentum $p_{K}<$ 800 MeV requirements, which are determined by particle identification and track reconstruction constraints.

The $\phi$(1020) production cross section is in agreement with the predictions of the MC generator tunes EPOS-LHC and PYTHIA6 DW. PYTHIA6 predictions using different tunes are observed to differ significantly. The cross section is also underestimated by PYTHIA8 and by Herwig++. This measurement can provide useful input for tuning and development of phenomenological models in order to improve MC generators.

## Footnotes

1 ATLAS uses a right-handed coordinate system with its origin at the nominal interaction point (IP) in the centre of the detector and the $z$-axis along the beam pipe. The $x$-axis points from the IP to the centre of the LHC ring, and the $y$-axis points upward. Cylindrical coordinates ($r,\phi$) are used in the transverse plane, $\phi$ being the azimuthal angle around the beam pipe. The pseudorapidity is defined in terms of the polar angle $\theta$ with respect to the beamline as $\eta = - \mathrm {ln[tan}(\theta /2)]$.
